# An Integrated Experimental and Theoretical Studies on the Corrosion Inhibition of Carbon Steel by Harmal Extracts

**DOI:** 10.3390/molecules27217250

**Published:** 2022-10-25

**Authors:** Hassan H. Hammud, Sarah A. Maache, Nasreen Al Otaibi, Nadeem S. Sheikh

**Affiliations:** 1 Department of Chemistry, College of Science, King Faisal University, Al-Ahsa 31982, Saudi Arabia; 2 Leading National Academy, Khobar Niagara College, Khobar 13244, Saudi Arabia; 3 Chemical Sciences, Faculty of Science, Universiti Brunei Darussalam, Jalan Tungku Link, Bandar Seri Begawan BE1410, Brunei

**Keywords:** harmal extract, electrochemical studies, anti-corrosion, DFT calculations

## Abstract

The corrosion inhibition effect of the three extracts from Harmal roots (HRE), leaves (HLE), and flowers (HFE) were studied for carbon steel corrosion inhibition in 0.25 M H_2_SO_4_ solution. The electrochemical impedance study indicated that the three types of extracts decreased corrosion effectively through a charge transfer mechanism. Harmal roots and leaf extracts showed inhibition values of 94.1% and 94.2%, while it was 88.7% for Harmal flower extract at the inhibitor concentration of 82.6 ppm. Potentiodynamic polarization data revealed that Harmal extracts acted through predominant cathodic type inhibition. Both the corrosion current density and corrosion rate decreased significantly in the presence of Harmal extracts compared to blank solution. The corrosion rate (mpy) value was 63.3, 86.1, and 180.7 for HRE, HLE, and HFE, respectively. The adsorption-free energy change Δ*G_ads_* (kJ·mol^−1^) values calculated from the Langmuir adsorption isotherm plots were for HRE (−35.08), HLE (−33.17), and HFE (−33.12). Thus, corrosion inhibition occurred due to the adsorption of Harmal extract on the carbon steel surface via the chemisorption mechanism. Moreover, a computational investigation using B3LYP/6-311G++(d,p) basis set in both gaseous and aqueous phases was performed for the major alkaloids (1–8) present in the Harmal extract.

## 1. Introduction

Corrosion [1] is a relentless and unavoidable process. However, the advancements in corrosion science have rendered a wide range of economically viable solutions. This ubiquitous natural phenomenon has a detrimental industrial impact and undoubtedly consumes billions of dollars annually. According to the statistics, the estimated cost of corrosion in China back in 2015 was ~USD 310 billion. This represented around 3.34% of the nation’s gross domestic product (GDP) [2]. More or less, similar statistics were reported for the corrosion cost in the United States of America [3]. The damaging exposure of metallic surfaces to air and moisture is considered one of the vast challenges on an industrial scale that can cause major economic losses [4]. Despite laser focus on technological advancements to prepare corrosion-resistant materials and develop efficient corrosion inhibitors, corrosion still poses a serious challenge for the scientific community. One of the most sustainable and efficient approaches to corrosion is an application of eco-friendly inhibitors [5,6,7,8] that produce minimal toxic waste. In this regard, the utility of organic substances as corrosion inhibitors is one of the most efficient protocols to mitigate metallic degradation [9]. This protocol is based on the adsorption of the inhibitor on the metallic surface to form a shielding layer that protects the metal from the adverse effect of corrosion. Quite recently, many classes of heterocyclic compounds have been extensively probed as efficient corrosion inhibitors. The presence of several functional groups such as hydroxyl, methoxy, amine, nitro, nitrile, carboxyl, ester, amide, and azo, among others, and multiple p-bonds acted as an adsorption point through which organic compounds bind with the metallic surfaces [10,11,12]. Additionally, polymeric structures [13] and materials [14] have been utilized as inhibitors, although these substances are relatively expensive with low environmental benign properties and polarity. Keeping all these aspects in consideration, current studies focused on exploring natural products as eco-friendly and cost-effective organic inhibitors, including plant extracts [15,16], oleochemicals [17], natural gums [18], and bio-surfactants [19,20].

**Figure 1:**
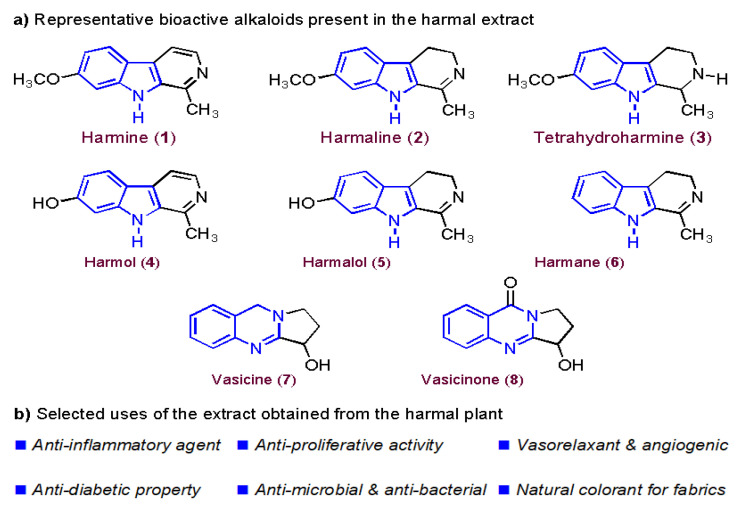
(**a**) Key bioactive indole-, quinazoline- and quinazolinone-based alkaloids present in the extract of the harmal plant and (**b**) generalized selected uses of the harmal extract [21].

Peganum harmal plant is one of the pharmaceutically significant herbs that primarily grows in the Asian regions [21]. It has been traditionally utilized as a natural medicine for treating tumors, infections, and inflammations in Greek, Iranian, Indian, and Chinese nations [22]. The significance of this plant is owed to the presence of active alkaloids, as shown in Figure 1a, which are concentrated in the plant’s seeds and roots [23]. Both harmine (**1**) and harmaline (**2**) exhibit, more or less, similar pharmacological effects, yet the latter has relatively less toxicity. Moreover, other indoles, quinazoline, and quinazolinone-based alkaloids (**3**–**8**) were also found to be active components in the harmal extracts with substantial medicinal utilities [24]. Specifically, harmine alkaloid (**1**) possesses anti-diabetic properties and has been used as a traditional medicine to treat diabetes. While harmaline (**2**), harman, and harmalol alkaloids act as vasorelaxants and angiogenic inhibitors to treat many cardiovascular diseases, including hypertension and bradycardia [25,26,27]. The extract of the harmal plant has also been used as an analgesic medicine to treat severe pain [28] and Parkinson’s disease [29]. In addition, the extract has anti-hallucination, anti-excitation, and anti-depression activities [30]. These active alkaloids have antitumor activity owing to their anti-proliferative effects that reduce the growth of colon cancer cells [31,32]. Other bioactive properties of the harmal extract include its application as an efficient antimicrobial and antibacterial agent against several types of bacteria, such as *Proteus vulgaris* and *Bacillus subtilis* [33,34]. Harmane (**6**) and vasicine (**7**) showed the most acetylcholinesterase inhibitor effect and can be used to treat Alzheimer’s disease [35]. In terms of respiratory disorders, vasicine (**7**) and vasicinone (**8**) have been reported to inhibit cough due to their bronchodilation activity [36]. In addition, the extract is also used at an industrial scale as a natural colorant for fabrics, especially wool, silk, and cotton [37].

DFT calculations are primarily used in inhibition studies as a fast, cost-effective tool to investigate the electron donor/acceptor regions in organic inhibitors. This protocol relies on providing a comprehensive study of chemical reactivity parameters to gain insight into the inhibition mechanism and, thus, support the experimental studies. Contrary to this, theoretical calculations warrant further investigations to accurately predict a relationship between the quantum chemical parameters and the corrosion inhibition efficiency due to limitations associated with the electron flow procedure [38,39].

Herein, we report the preparation of Harmal extracts by sonication from three portions of the plant roots, leaves, and flowers. The extracts were tested as corrosion inhibitors of carbon steel (C-steel) in dilute sulfuric acid solution using impedance and potentiodynamic polarization techniques. Harmal plant extract is an eco-friendly corrosion inhibitor because it is less toxic, biodegradable, highly efficient, available, renewable, and inexpensive. Moreover, the constituents of Harmal extracts have nitrogen and oxygen functional groups that can act as adsorption centers to the metal surface, Figure 1a. Additionally, a computational investigation for the potent bioactive alkaloids present in the Harmal extract has been delineated.

## 2. Experimental

### 2.1. Materials

#### 2.1.1. Preparation of Harmal Parts Extracts

Harmal plants were collected from Alhasa in the east regain of Saudi Arabia. Leave root and flower portions were separated, dried in the shade, and then ground to powder. Then, 100 g of powder from each plant portion was added to 100 mL ethanol in the bottom flask. The solution was then continuously sonicated for 1 h at 80 kHz using a POWER SONIC405 [40]. The green extract was filtered then the solvent was removed using a rotary evaporator. The obtained residue was further dried in an air oven at 50 °C for several hours. About one gram of green powder was obtained. A 0.100 g of the powder was dissolved in 10.00 mL methanol to obtain a 10,000 ppm stock solution for each plant portion. Then they were labeled as Harmal roots extract (HRE), Harmal leaves extract (HLE), and Harmal flowers extract (HFE). The green extract solutions were later used as corrosion inhibitors for C-steel in 0.25M H_2_SO_4_ solution.

#### 2.1.2. Working Electrodes

A cylindrical carbon steel (C-steel) electrode with an exposed area of 0.5027 cm^2^ was used as the working electrode. The following is the chemical composition of carbon steel rod: % weight (wt%) C, 0.164; S, 0.001; Mn, 0.710; P, 0.0005; Si, 0.26, Ni, 0.123; Cr, 0.041; balance Fe. The C-steel working electrode was buffed with a series of silicon carbide papers from 600 to 1200 before the beginning of the electrochemical measurements.

#### 2.1.3. Electrolytes

The working electrode was immersed in 0.25 M H_2_SO_4_ solution containing 0, 20.8, 29.1, 41.5, 62.1, and 82.6 ppm of the Harmal extract. The standard solutions were prepared by diluting the stock solution (10,000 ppm) with the corresponding dilute water portions.

### 2.2. Methods

#### 2.2.1. Electrochemical Experiment

Impedance study (EIS) and potentiodynamic polarization study (PDP) measurements were carried out using (Gamry, reference 600 Potentiostat/Galvanostat/ZRA, Warminster, PA, USA) and Gamry software v7.07. The cell used consisted of platinum (Pt) wire auxiliary electrode and a saturated silver reference electrode (Ag/AgCl) electrode. A C-steel rod was used as a working electrode, as described above. Before the start of the impedance experiment, the steady-state open circuit potential E_OCP_ was measured by immersing the electrodes in the corrosion solution for 60 min with the indication 10 mV disturbance capacity [41].

Potentiodynamic polarization measurements were carried out in the direction from the cathode to the anode from 500 mV versus the resting potential at a scan rate of 5 mV/s. The percent inhibition *IE*% and fractions of surface coverage (*θ*) were evaluated from the following Equations (1) and (2) [42]:

(1) $$IE\%=\left(1-\frac{I\;\mathrm{corr}\left(i\right)}{I\;\mathrm{corr}\left(0\right)}\right)\times 100$$

(2) $$\theta =1-\frac{I\mathrm{corr}\left(i\right)}{I\mathrm{corr}\left(0\right)}$$

where, *I*_corr(0)_ and *I*_corr(i)_ are the corrosion current density of the steel specimen (mA.cm^−2^) for the uninhibited and inhibited solutions, respectively.

Electrochemical impedance studies (EIS) were carried out at a potential amplitude of 10 mV, peak to peak (AC signal) in frequencies ranging from 100 kHz to 0.1 Hz.

The obtained impedance results were represented as Nyquist and Bode plots [41]. The following modified Equation (3) was used to calculate the inhibition efficiency (IE%) from the Nyquist plot [42].

(3) $$IE\%=1-\left(\frac{Rp\left(0\right)}{Rp\left(i\right)}\right)\times 100$$

The polarization resistance *R*_p_ = *R*_ct_ + *R*_f_, where *R*_ct_ is the charge transfer resistance of C-steel and *R*_f_ is the coating resistance. (0) indicate a blank solution, and (i) indicates an inhibitor solution. Moreover, the surface coverage (*θ*) was calculated using the following Equation (4):

(4) $$\theta =1-\frac{Rp\left(0\right)}{Rp\left(i\right)}$$

#### 2.2.2. Computational Details

Density functional theory (DFT) [43] calculations were performed using Gaussian 09 (revision E.01) [44], and the Gaussview [45] was used to generate input geometries and visualize the output structures. Geometry optimizations and frequency calculations for the key constituents 1–8 present in the extract of Harmal leaves were performed using the B3LYP functional [46,47,48] with the 6-311++G(d,p) basis set [49]. The calculations were performed in the gaseous phase. However, for comparative purposes and to model the solvation effect, the calculations were carried out in an aqueous medium as well by applying the most commonly used integral equation formalism (IEF) version of the polarized continuum model (PCM) [50,51]. This is essential as the electrochemical corrosion phenomenon takes place in a liquid phase. All stationary points were characterized as minima based on normal vibrational mode analysis. Thermal corrections were computed from unscaled frequencies, assuming a standard state of 298.15 K and 1 atm. The structures described herein are the lowest energy-optimized conformers.

## 3. Results and Discussions

### 3.1. Corrosion Inhibition Study

The open-circuit potential, *E*_ocp_, for each of the experiments conducted was first examined for 60 min before applying an electrochemical impedance study (EIS) to obtain a steady current reading on the carbon steel surface [52].

The open circuit potential *E*_ocp_ plots versus time (s) of the C-steel with and without the addition of different concentrations of (a) Harmal Roots extracts, (b) Harmal Leaves extracts, and (c) Harmal Flowers extracts in 0.25M H_2_SO_4_ are shown in Supplementary Figure S1.

#### 3.1.1. Electrochemical Impedance Study (EIS)

**Figure 2:**
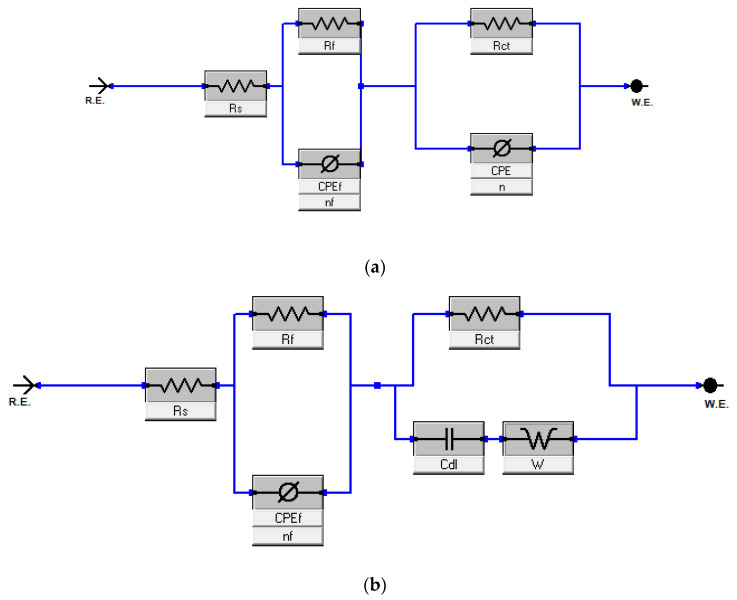
Equivalent circuits (**a**) Model 1 and (**b**) Model 2 used to fit the impedance data of the Harmal extracts.

**Figure 3:**
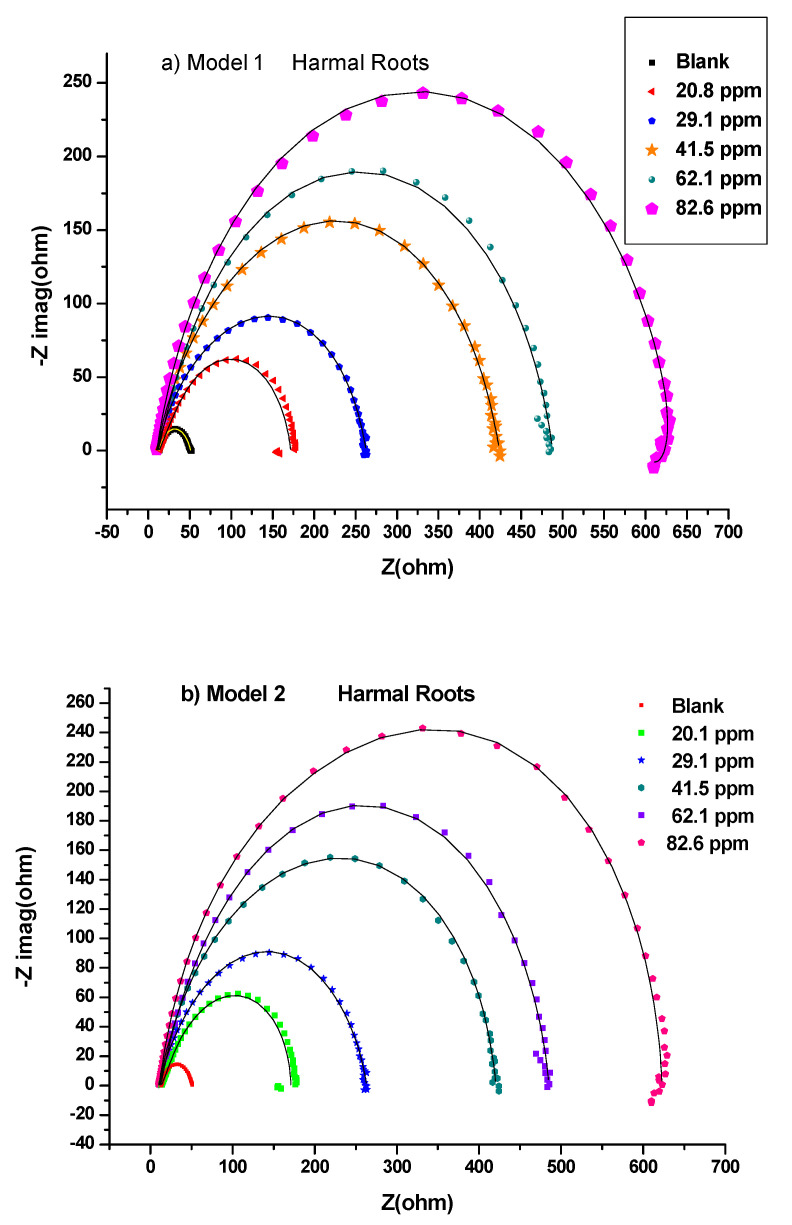
Nyquist plots for all concentrations of Harmal roots extract inhibitors in 0.25 M H_2_SO_4_ at 298 K fitted by (**a**) Model 1 and (**b**) Model 2.

**Figure 4:**
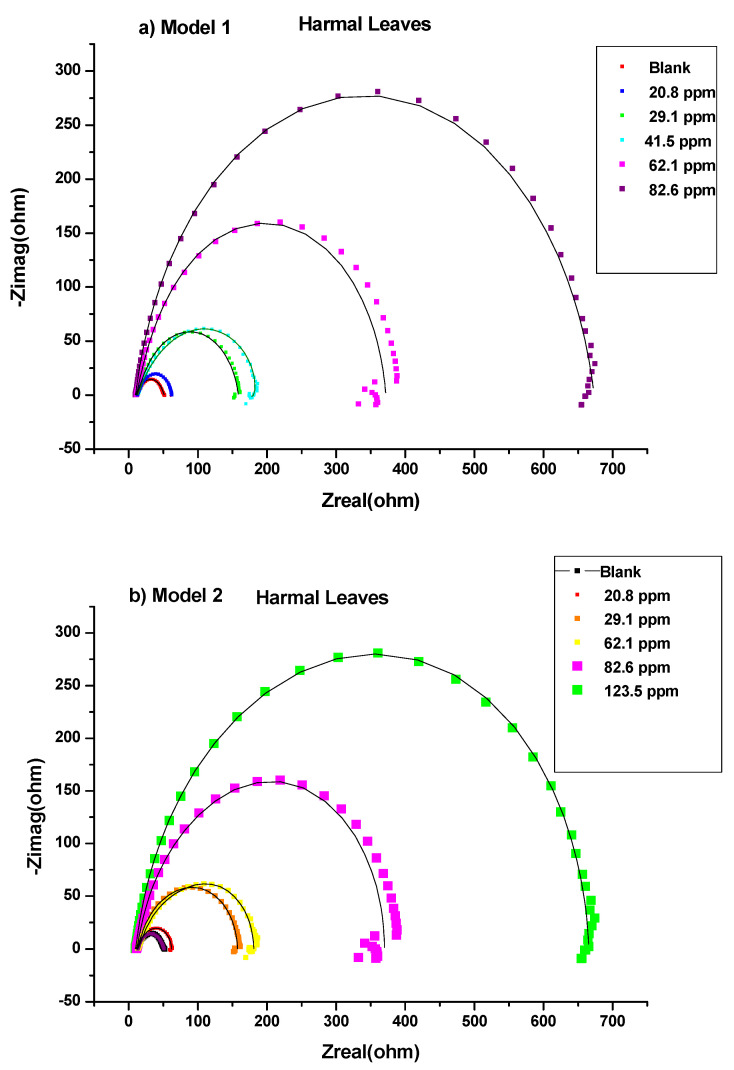
Nyquist plots for all concentrations of Harmal leaves extract inhibitors in 0.25 M H_2_SO_4_ at 298 K fitted by (**a**) Model 1 and (**b**) Model 2.

**Figure 5:**
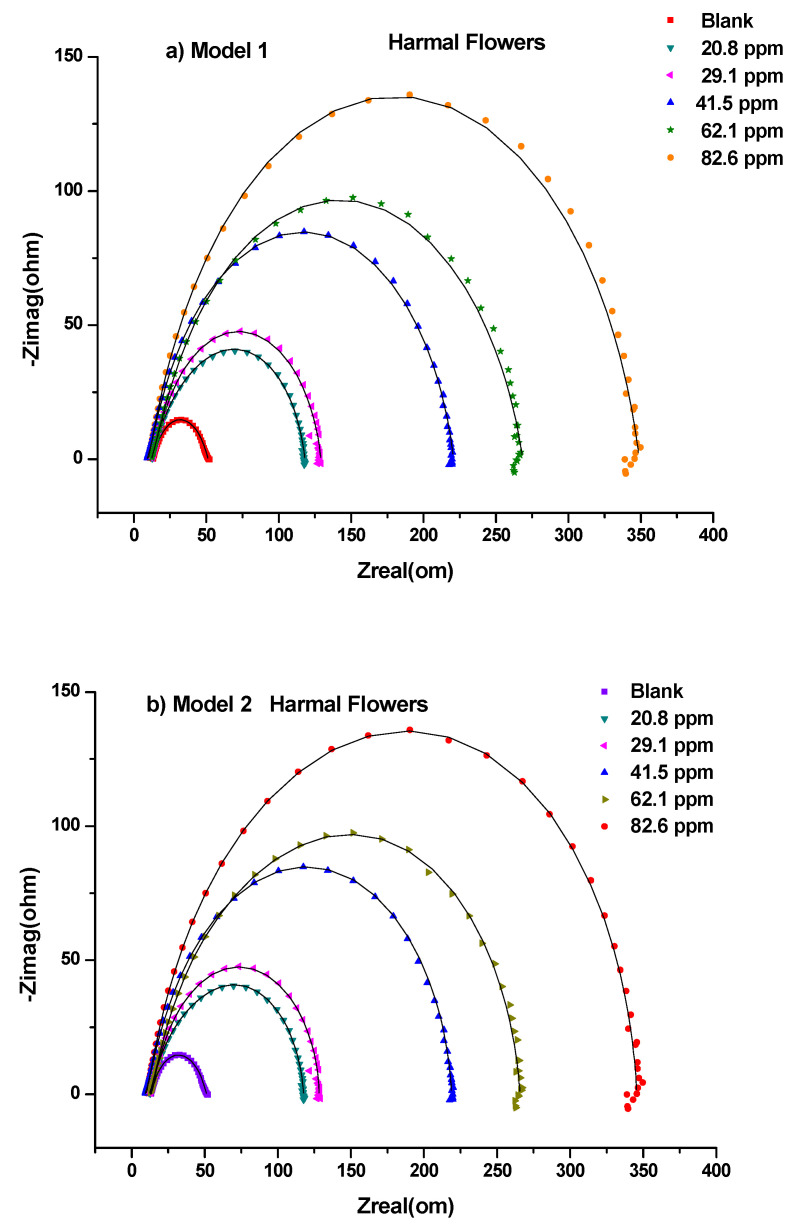
Nyquist plots for all concentrations of Harmal flower extract inhibitors in 0.25 M H_2_SO_4_ at 298 K fitted by (**a**) Model 1 and (**b**) Model 2.

This study was performed to investigate the amount of current flow and the resistance values occurring on the carbon steel surface with and without the presence of inhibitors [52]. All the processes involved in the electrical response of the system were fitted against the equivalent electrical circuits (EEC) model 1 (Figure 2a) and model 2 (Figure 2b). Nyquist plots are shown in Figure 3 for HRE, Figure 4 for HLE, and Figure 5 for HFE, with (a) for the fitting by model 1 and (b) for the fitting by model 2.

Thus, the equivalent electrical circuit models 1 and 2 with two-time constants were used. They gave a good fitting of impedance data for the blank and the solutions of all ranges of concentrations of Harmal extract roots (Table 1 and Table 2), leaves (Table 3 and Table 4), and flowers (Table 5 and Table 6), respectively.

In general, each electrochemical process in the circuit should be represented by a separate distinct semicircle in the Nyquist plot [42]. But, the first semicircle is not observed due to the high-frequency limit [53]. The fitted model 1 consists of two circuits connected in series and contains solution resistance (*R*_s_), film resistance (*R*_f_), charge-transfer resistance (*R*_ct_), constant phase element of the film (CPE_f_), and the double layer (CPE_dl_). The double-layer capacitance (*C*_dl_) can also be calculated from the values of *R*_ct_, the impedance of CPE_dl_ (*Z*_CPE_), and the exponents of CPE_dl_ (*n*) [41]. The data fitted by model 1 is shown in Table 1, Table 3, and Table 5; Figure 3a, Figure 4a, and Figure 5a for HRE, HLE, and HFE, respectively.

The solution resistance (*R*_s_) describes the ohmic resistance, while the charge transfer resistance (*R*_ct_) represents the inhibitor’s resistance towards oxidation of the metal surface, and it is inversely proportional to the corrosion rate [53]. *R*_f_ or coating resistance (*R*_c_) is referred to as an intact film/coat (isolation layer), which provides good protective performance [54].

The EEC model 2 (Figure 2b) was applied, which added a diffusion component to model 1 (Figure 2a) to fit the experimental data. If the diffusion region is close to the inhibitor film/metal interface, the mass transfer of reactive species is decreased, resulting in a Warburg impedance *W* [41,54]. Moreover, a constant phase element (CPE) of model 1 is replaced by a pure double layer capacitor (Cdl) in model 2 to fit the semicircle shape of the Nyquist plot. The results of fitting the data to model 2 are shown in Table 2, Table 4, and Table 6.

A careful investigation of Nyquist plots shows that model 2 showed a slightly better fitting than model 1 when higher concentrations of 62.1 ppm and 82.6 ppm were used, Figure 3, Figure 4 and Figure 5. This indicates that the mass transfer of reactive species at higher concentrations was slowed down next to the inhibitor film/metal interface resulting in a Warburg impedance *W.*

The higher the inhibitor concentrations, the higher the diameter of the Nyquist plots Figure 3, Figure 4 and Figure 5 [55]. Thus, the higher the polarization resistance values *R*_p_ *= R*_ct_ + *R*_f_, Table 1 (HRE), Table 3 (HLE) and Table 5 (HFE) using model 1 and Table 2 (HRE), Table 4 (HLE) and Table 6 (HFE) using model 2. This caused an increase in the inhibition efficiency of the inhibitor. This could correspond to the strengthening of the inhibitive film on the carbon steel surface [56].

According to Table 1, Table 2, Table 3, Table 4, Table 5 and Table 6, Harmal roots and leaves extracts showed a maximum inhibition value averaged from both models, 94.1% and 94.2% at the concentration of 82.6 ppm, which are higher compared to 88.7% for Harmal flowers extract at the same concentration. The maintained semicircle shapes of the Nyquist plots over the experiments revealed that the corrosion inhibition process occurred through a charge transfer mechanism [52]. The increase in *R*_ct_ systematically and the decrease in *C*_dl_ values with few exceptions upon adding Harmal extracts of various portions of roots, leaves, and flowers indicated a reduction in the corrosion rate. This is because of the replacement of water molecules on the working electrode surface by the inhibitor’s protective films Table 1–6.

##### Bode and Bode Phase Angle

**Figure 6:**
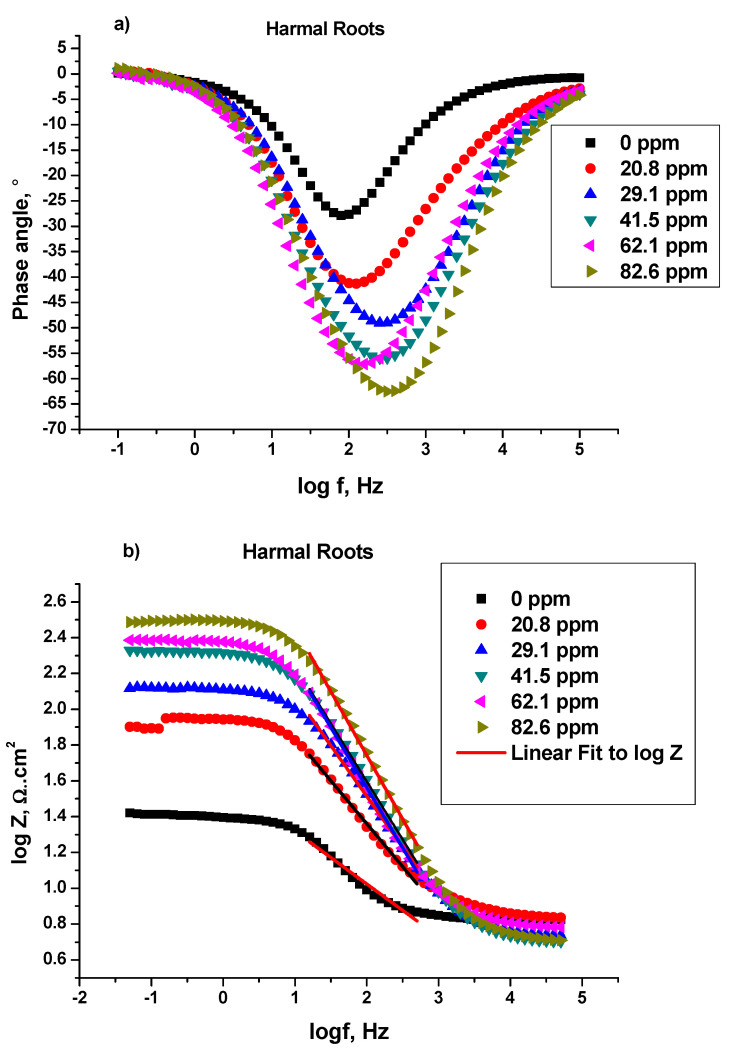
Bode phase (**a**) and Bode (**b**) plots of a C-steel electrode in 0.25 M H_2_SO_4_ solution with and without different concentrations of Harmal roots extract.

**Figure 7:**
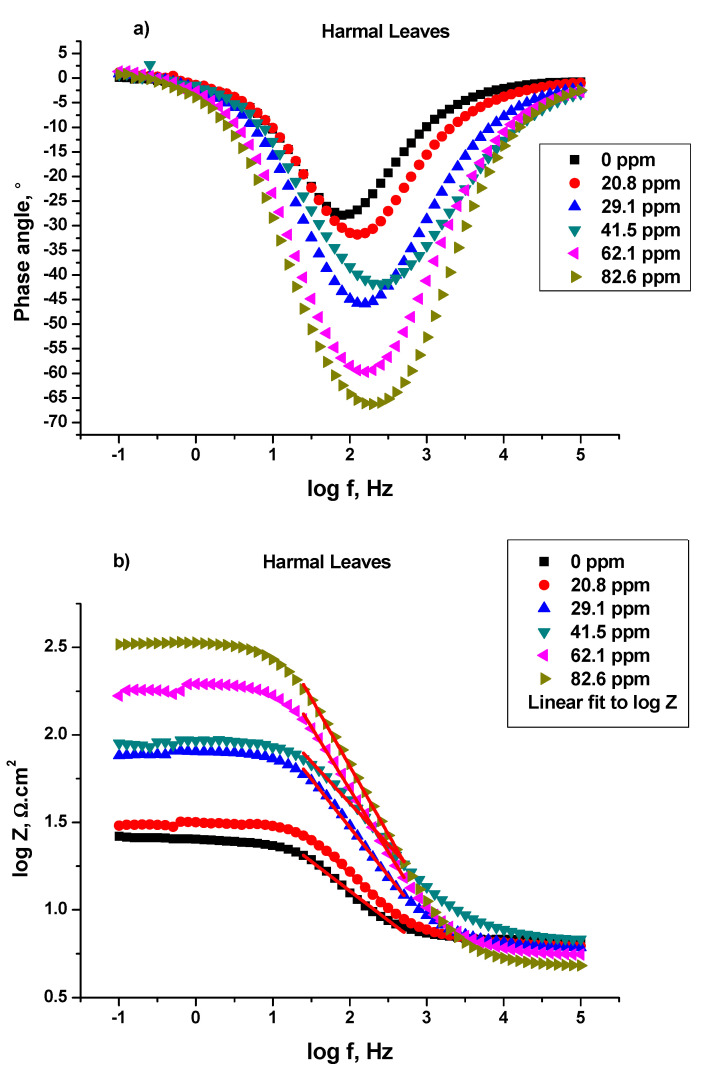
Bode phase (**a**) and Bode (**b**) plots of a C-steel electrode in 0.25 M H_2_SO_4_ solution with and without different concentrations of leaf extract.

**Figure 8:**
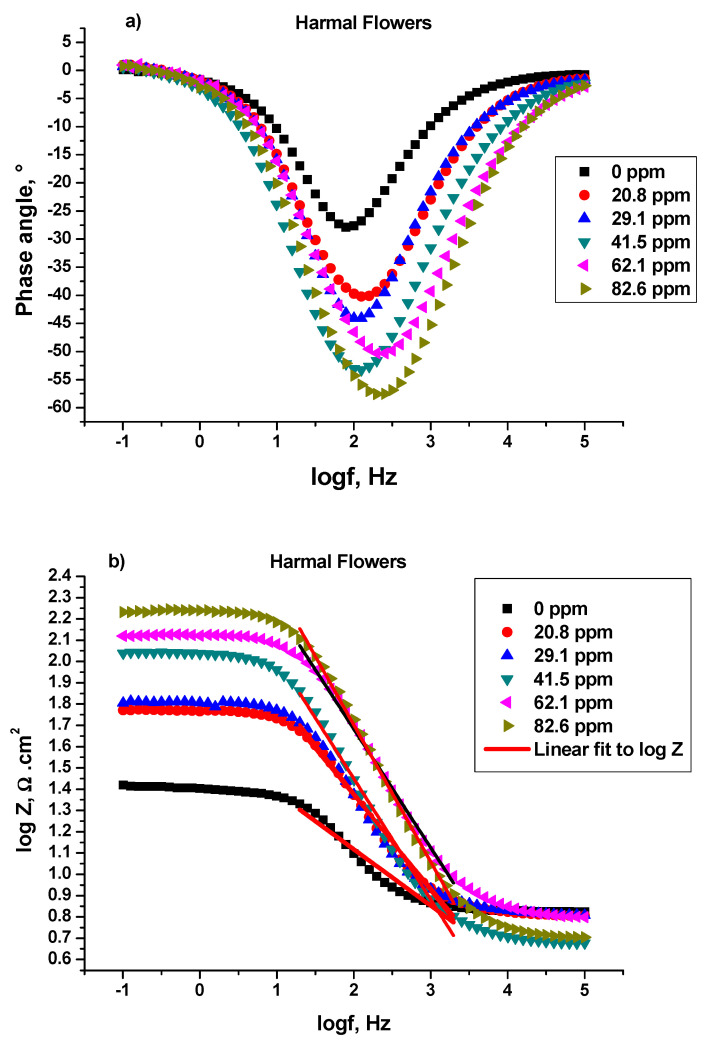
Bode phase (**a**) and Bode (**b**) plots of a C-steel electrode in 0.25 M H_2_SO_4_ solution with and without different concentrations of Harmal flower extract.

Bode and Bode phase plots: log (imaginary part of impedance) (log |Z| (ohm)), and phase angle (°) were plotted against log frequency (log*f* (Hz)) respectively, Figure 6, Figure 7 and Figure 8, for C-steel in 0.25 M H_2_SO_4_ in the absence and presence of Harmal extracts [41]. Figure 6a, 7a, and 8a show the log Bode phase angle (°) plotted against the log*f* (Hz) for the inhibitor HRE, HLE, and HFE, respectively.

It is obvious that increasing the Harmal extract concentration causes more negative values of phase angle at the intermediate frequency (peak), and thus the inhibitive behavior increases [41,42]. In fact, the phase angle values increased from −27.87° for the blank solution to −62.52° (HRE), −66.28° (HLE), and −57.58° (HFE) for the inhibitor solution (82.6 ppm), Table 7.

The α values represent the surface irregularities for C-steel. They were calculated for the Harmal extracts from the slope of the linear region of log|Z| versus the log*f* plots [42]; (HRE) Figure 6b, (HLE) Figure 7b, and (HFE) Figure 8b; Z (ohms) being the imaginary part of impedance and *f* (Hz) is the frequency. Hypothetically, the value of α is equal to −1 for an ideal capacitor. In contrast, the coarseness of the C-steel surface occurs for α values less than −1. It can be seen that the presence of Harmal extracts increased the α value from −0.3635 (0 ppm) to −0.7190 (HRE), −0.7671 (HLE), and −0.6571 (HFE) for the Harmal extract (82.6 ppm), Table 7. Thus, the decrease of heterogeneity of the C-steel surface occurred as a result of the inhibition of Harmal extracts.

#### 3.1.2. Potentiodynamic Polarization Study (PDP)

**Figure 9:**
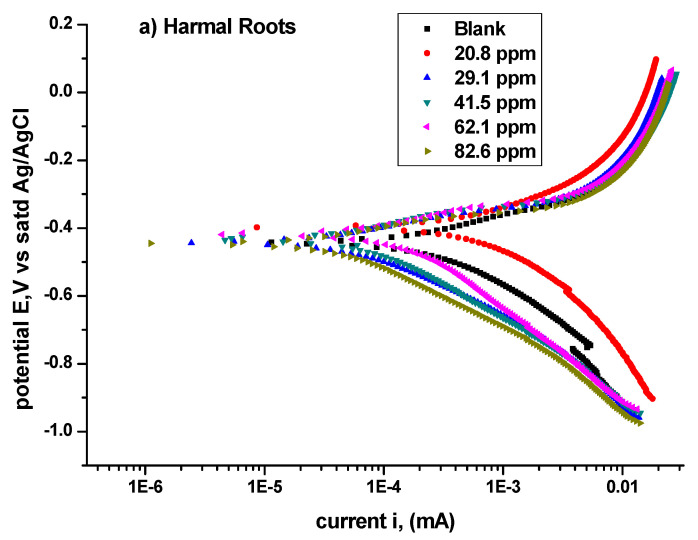

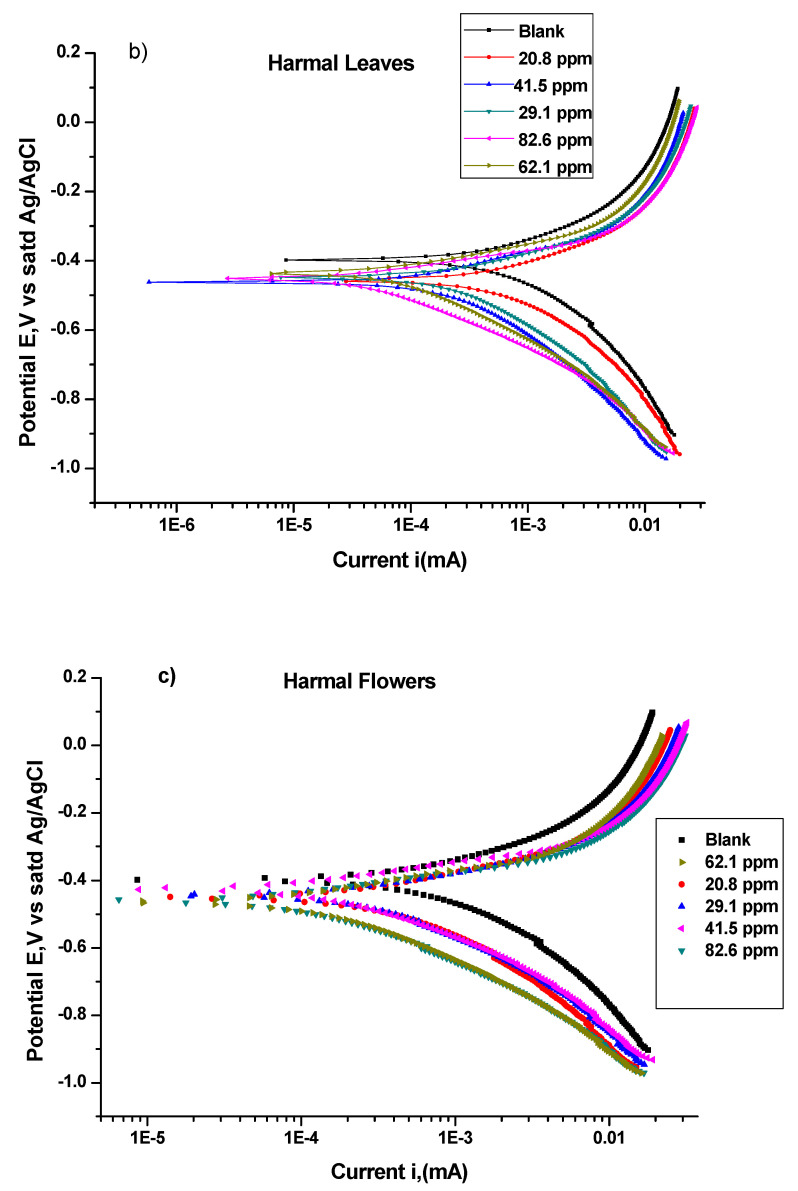
Tafel plots for all concentrations of (**a**) Harmal roots, (**b**) Harmal leaves, and (**c**) Harmal flowers extract inhibitors in 0.25M H_2_SO_4_ at 298 K.

Figure 9a–c shows the Tafel polarization curves obtained for carbon steel in the absence and presence of Harmal part extracts roots, leaves, and flowers, respectively, in 0.25 M H_2_SO_4_ at room temperature.

The analysis of the Tafel polarization curves is computed using the Gamry software v7.07, and the results are presented in Table 8 (HRE), Table 9 (HLE), and Table 10 (HFE). The equilibrium corrosion potential (*E*_corr_) values shifted to more negative values for the inhibitor in comparison with the blank solution. But the potential *E*_corr_ does not follow a consistent increase with increasing concentration of inhibitors. This was also observed in other reported works [42,56]. The inhibitors could be classified as mixed-type inhibitors [52]. But there is a consistent *I*_corr_ current decrease with increased concentration, which clearly indicates the enhancement of inhibition. Thus, the current values are more reliable than the potential values to evaluate inhibition efficiency.

*I*_corr_ (μA/cm^2^) for the diluted sulfuric acid blank is 3401.63, while for 82.6 ppm solution of extract, it is 138.65 (HRE), 188.38 (HLE), and 395.86 (HFE). The greatest decrease in current occurred for HRE. The organic constituents of Harmal part extracts probably adsorbed on the carbon steel surface and formed an insulating film which caused decreasing in corrosion current values [57]. The decrease in the electrical current (*I*) flow is due to a lowering in the number of electrons being transferred across the metal surface. This suggests that the metal dissolution process (oxidation of iron) was hindered and that the Harmal extracts act as anodic inhibitors [52].

Accordingly, the corrosion rate in mils penetration per year (mpy) decreased from 1554.0 for the blank solution to 63.34 for HRE, 86.1 for HLE, and 180.7 HFE extracts, at an 82.6 ppm inhibitor concentration. Again, the greatest decrease in corrosion rate also occurred for HRE, and the corrosion rate followed the order HRE < HLE < HFE. This indicated that the extracts’ organic constituents inhibited carbon steel’s corrosion process in a dilute H_2_SO_4_ solution. Moreover, it is clear from the Tafel plots in Figure 9a–c that the addition of an inhibitor effectively suppressed cathodic reactions. This suggests that Harmal extracts functioned as a cathodic inhibitor. In addition, the corrosion parameters such as *E*_corr_, *I*corr, slope of anodic (*β*a) and cathodic (*β*c), and inhibition efficiency values are listed in Table 8, Table 9 and Table 10. The addition of inhibitor decreased the magnitude of *β*a and *β*c, indicating that the extracts acted as an anodic and cathodic inhibitor of mild steel samples in H_2_SO_4_ solutions [56].

On the other hand, the calculated % inhibition efficiency %*IE* from the Tafel curves for the highest inhibitor concentration (82.6 ppm) was 95.9% for HRE, 94.5% for HLE, and 88.4% for HFE. The obtained *%IE* using polarization studies are in close agreement with those obtained using the impedance studies. In conclusion, the inhibitor strength is HRE > HLE > HFE.

In the present work, we have studied three extracts, Harmal roots, leaves, and flowers, instead of only the Harmal leaves extract previously reported [41]. We have concluded that all three types of extracts can be considered efficient inhibitors. The immersion time of C-steel in 0.25M H_2_SO_4_ solution in the present work was 60 min compared to 15 min previously. The inhibition of 94.2% in the present work for Harmal leaves extract at 82.6 ppm is much higher than the previously reported 59.7% at 82.6 ppm. One concludes that the longer the time of exposure to inhibitors solutions, the higher the *IE%*.

### 3.2. Adsorption Isotherms

**Figure 10:**
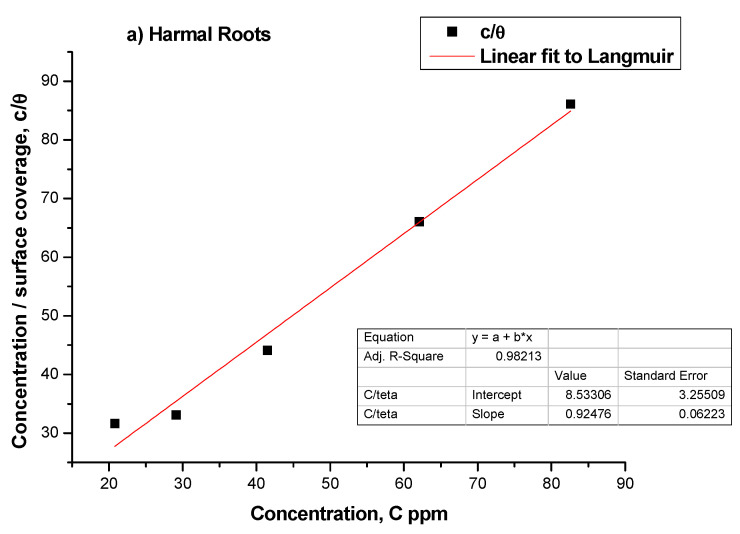

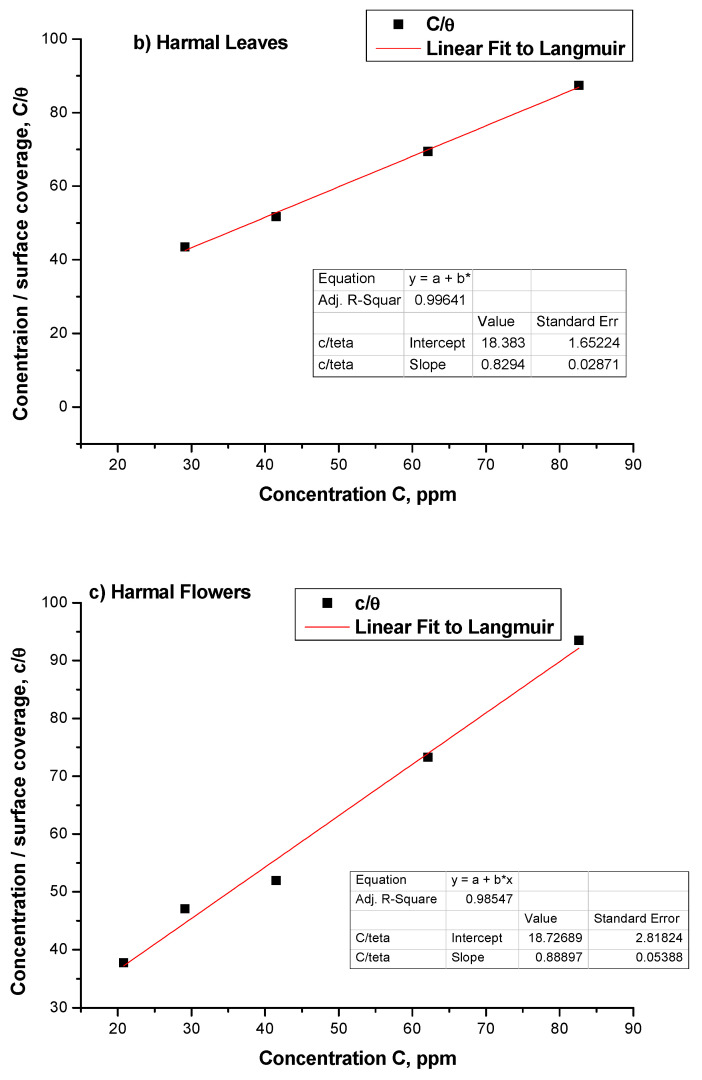

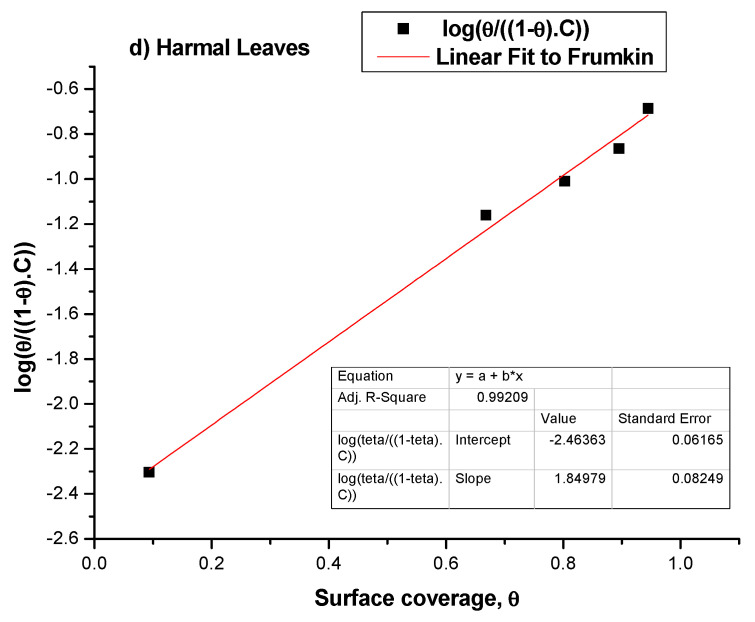
Langmuir adsorption isotherm of Harmal (**a**) roots, (**b**) leaves, and (**c**) flower extract, and (**d**) Frumkin adsorption isotherm of Harmal leaves extract; for all concentrations on carbon steel electrode in 0.25M H_2_SO_4_ at 298 K.

In order to study the adsorption behavior of Harmal extracts in H_2_SO_4_ solution, several isotherm models were implemented using the inhibition values obtained from polarization studies. These are Langmuir, Frumkin, and Temkin [41]. However, the best fit was obtained for the Langmuir isotherm model for all three extracts Figure 10a–c, and additionally, Frumkin also fitted the data of the HLE extract, Figure 10d.

The Langmuir isotherm model monitors the variation of adsorption coefficient *K*_ads_ with a concentration of inhibitor C according to the following relationship Equation (5) [56]:

c/*θ* = c + 1/*K*_ads_ (5)

The adsorption parameters, regression coefficient *R*^2^, *K*_ads_, and slope values are obtained by straight line fitting between C/*θ* and C, Figure 10a (HRE), b (HLE), and c (HFE). The *R*^2^ coefficient values are all ≥0.98. It is obvious that the slope values are close to 1.0, as expected from the Langmuir model Equation. According to the Langmuir adsorption isotherm, the tested inhibitor molecules have a typical adsorption site at the metal/solution interface with no interaction with the other molecules adsorbed.

The fitting of the Temkin model was tested by plotting log(*θ*/C) versus *θ*. The *R*^2^ values obtained from the straight line for HRE, HLE, and HFE are 0.4934, 0.5492, and 0.8343, respectively. Thus, the Temkin model is not an acceptable model in all cases. In contrast, the Frumkin model plot of log[*θ*/(1 − *θ*)·C)] versus *θ* gave *R*^2^ values of 0.9086, 0.9929, and 0.8699 for HRE, HLE, and HFE (Figure 10d). These values are also a little smaller than the corresponding *R^2^* values obtained from the Langmuir model, Figure 10a–c. Thus, the Langmuir model is the best model to represent the adsorption of Harmal molecules of the three extracts onto C-steel.

The adsorption equilibrium constant (*K_ads_*) was calculated using the inverse of the intercept of the Langmuir plot. In all the adsorption isotherm plots, the concentration used was ppm. The obtained *K*_ads_ (ppm^−1^) values from the Langmuir isotherm plots were just converted to *K*_ads_ (mol/L)^−1^. The latter values were used to calculate Δ*G*°_ads_. The values of *K*_ads_ for HRE, HLE, and HFE were 24.61 × 10^3^ M^−1^, 11.42 × 10^3^ M^−1^, and 11.21 × 10^3^ M^−1^, respectively. The standard free energy of adsorption Δ*G*^0^_ads_ can be calculated from *K*_ads_ using Equation 6 [40]:

Δ*G*^0^*_ads_* = −*RT* ln(55.5*K*_ads_) (6)

where *R* is the gas constant (8.314 J mol K^−1^), and *T* is the absolute temperature (*K*). The obtained values for Δ*G*^0^*_ads_* are −35.08, −33.17, and −33.12 kJ/mol for HRE, HLE, and HFE, respectively; indicating that the adsorption mechanism of the inhibitors onto the C-steel in 0.25 M H_2_SO_4_ solution involved chemisorption adsorption [42]. Finally, it is noted that HRE had the highest value of *K_ads_* and Δ*G*^0^*_ads_* and thus exhibited the most effective corrosion inhibition.

### 3.3. Inhibition Mechanism

Corrosion inhibition of C-steel in sulfuric acid solution by different Harmal organic molecules present in the three extracts can be explained on the basis of molecular adsorption. The compounds inhibit corrosion by blocking cathodic sites. In a weak acidic solution, the Harmal constituents can exist as neutral or protonated species (by protonation of the nitrogen atom by H^+^ of sulfuric acid).

The neutral species are chemically adsorbed on steel, while protonated species are physically adsorbed on the carbon steel forming a protective film. This causes a decrease in the exposed surface area of steel in contact with the electrolyte solution and thus causes a decrease in the evolution of hydrogen gas.

**Figure 11:**
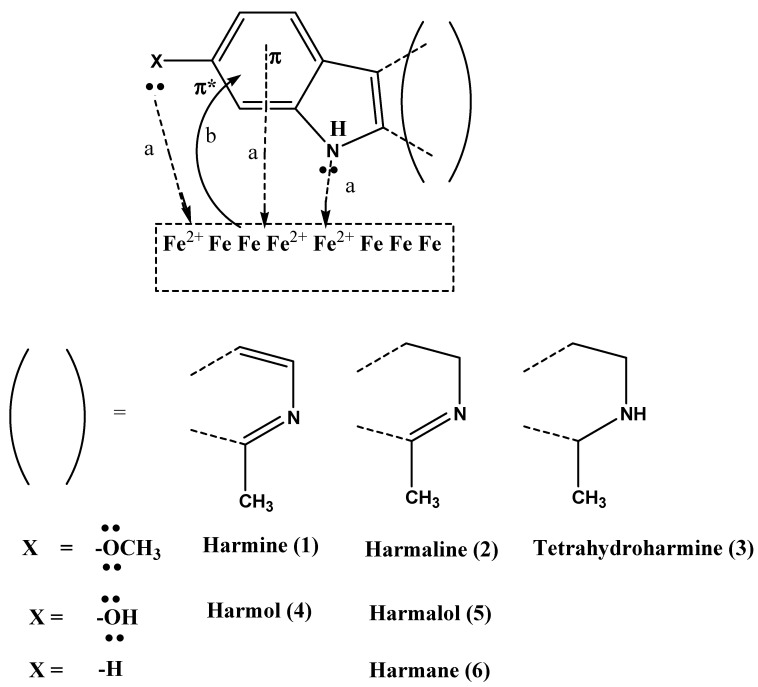
Inhibition mechanism based on chemical adsorption of the Harmal molecules from Harmal plant extracts HRE, HLE, and HFE. (**a**) σ bonds and (**b**) π back bonds are shown for the coordinate bonds between the Harmal molecules and Fe or Fe^2+^ on the C-steel surface.

The chemical adsorption of Harmal constituents occurs through the formation of coordination bonds between electron-rich functional groups orbitals of Harmal molecules with empty d- orbital of the Fe atom or Fe^2+^ ion, Figure 11. π electrons of imines >C=N, and aromatic rings and lone pair of electrons of different hetero nitrogen and oxygen atoms from Harmal molecules can be shared with iron orbitals. Chemical adsorption onto the C-steel surface can also occur through π-electrons back donation from Fe d_t2g_ orbital onto empty π* orbital of aromatic rings of the Hamal molecules, Figure 11.

The physical adsorption of the Harmal molecules can occur via electrostatic interactions between the protonated Harmal molecules and the negatively charged steel. This is because the SO_4_^2−^ anion from H_2_SO_4_ is attracted to a positively charged steel surface containing (Fe^2+^) via electrostatic interactions, rendering the surface of the C-steel negatively charged. Therefore, both the physical and chemical adsorption of Harmal molecules favor stronger adsorption and inhibit corrosion by blocking the active site of the iron surface.

**Figure 12:**
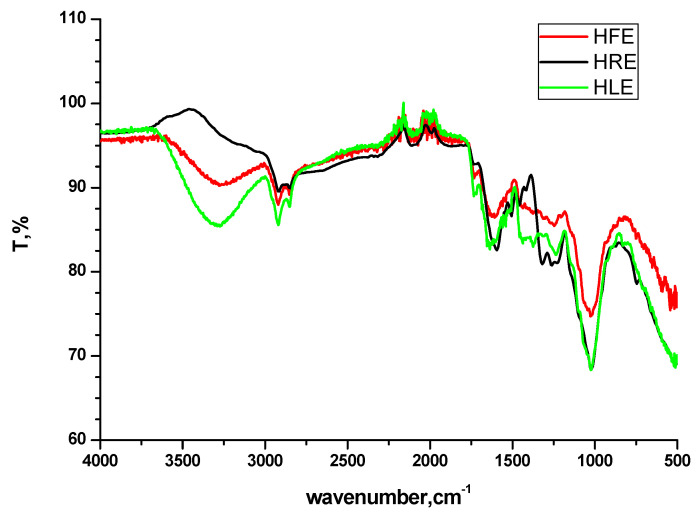
FTIR spectra of the extracts of Harmal roots (HRE), Harmal leaves (HLE), and Harmal flowers (HFE).

The % inhibitor efficiency calculated from impedance and Tafel studies ranges from about 90% to 95% for the three extracts in the order HRE > HLE > HFE. This indicates that the extracts from three parts of the Harmal plants can be used as corrosion inhibitors. Thus, this study indicates that most of the Harmal plants can be collected, grounded, extracted, and used as an inhibitor. Therefore, the production yield to be used from Harmal plant is much higher than focusing on only one extract from leaves; thus, it is of greater economic value for industrial application. Moreover, from FTIR studies (Figure 12), there is an indication that the three extracts contain mainly the same electron-rich functional groups that promote inhibition, such as hydroxyl, amine, imine, alkoxy, and aromatic rings. The stretching vibrations (cm^−1^) occur for: N-H and O-H at 3278 and 3200 cm^−1^, C-H aromatic at 2925 and 2840, O-H carboxylic at 2632, >C=O stretch at 1732 cm^−1^, >C=N and >C=C< alkene at 1640 and aromatic at 1440–1586, >C-O at 1024 and 1250. The bending vibration (cm^−1^) occurs for C-H aromatic at 748.

### 3.4. Comparative Studies of Inhibition Efficiency IE%

The corrosion inhibition efficiency of steel by Harmal roots, leaves, and flower extracts in sulfuric acid media was compared with other ones reported in the literature, Table 11. The results showed that the three Harmal extracts studied at a low concentration of 83 ppm with high *IE*% (93%), averaged from the three extracts, are more effective towards the inhibition of C-steel corrosion compared to other reported ones: Date palm seed (2000 ppm, 91%) [58], tea tree (2250 ppm, 78.6%) [59], Ginkgo leaf extract (200 ppm, 90.0%) [60], and Brassica oleracea L (300 ppm, 92.7%) [61].

### 3.5. Surface Morphology

**Figure 13:**
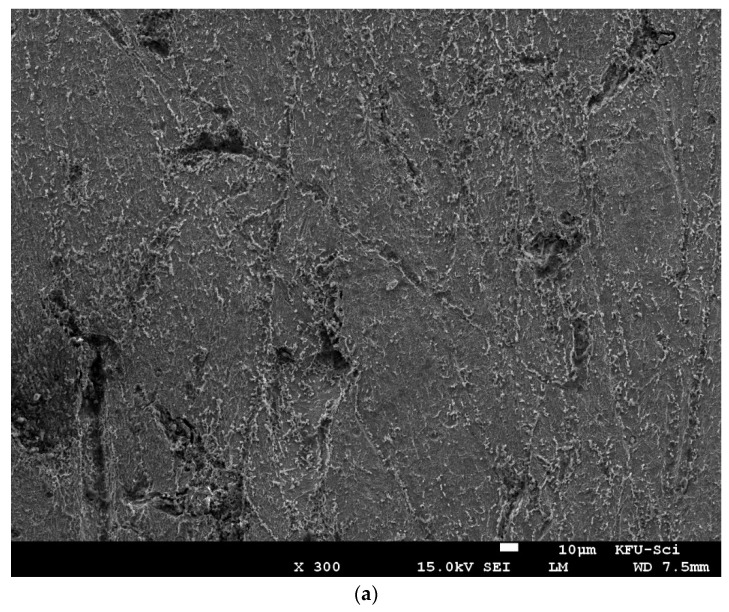

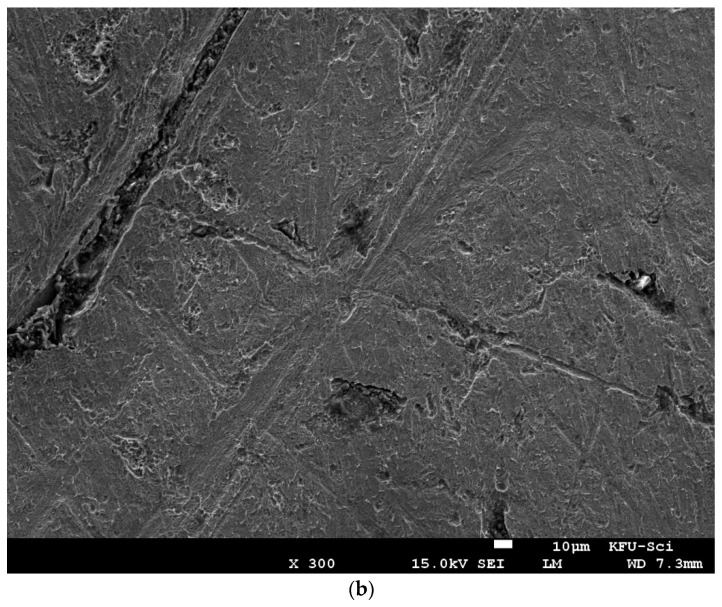
SEM images of C-steel immersed for 3 h in (**a**) free 0.25 M H_2_SO_4_ and (**b**) 0.25M H_2_SO_4_ containing 300 ppm of Harmal leaf extract (HLE).

Figure 13 shows SEM micrographs of C-steel immersed for 3 h in (a) free 0.25 M H_2_SO_4_ and (b) 0.25 M H_2_SO_4_ containing 300 ppm of Harmal leaf extracts HLE. The morphology in Figure 13a shows a rough surface containing numerous white particles of iron oxides pointing out from the surface, which characterizes the uniform corrosion of C-steel in sulfuric acid solution. While in the presence of HLE extracts (Figure 13b), a smoother surface can be observed because of the formation of the eco-friendly inhibitor’s protective film on the metal surface. This finding may confirm that the presence of Harmal extracts molecules improves the inhibition efficiency of the C-steel.

### 3.6. Computational Studies

**Figure 14:**
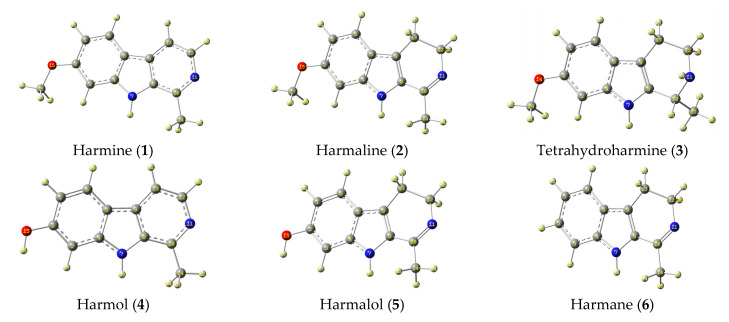

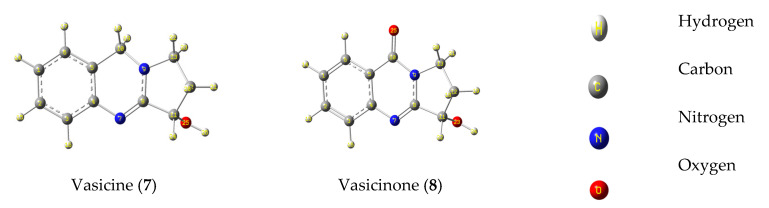
Optimized structures of the stable conformers for the neutral form of organic molecules **1**–**8** present in the extract of Harmal leaves computed at the B3LYP/6-311G++(d,p) in water using the PCM solvation model [49].

The optimized structure, depicted in Figure 14, represents the stable conformers of eight key organic compounds present in the Harmal extract. All these alkaloids present in the Harmal extract have structurally related units; however, they vary due to the presence of methoxy and hydroxyl groups. Another noticeable feature is the presence of the carbonyl group in alkaloid 8, which may impact its behavior and display dissimilar values of electronic properties than structurally related natural alkaloid 7. Moreover, the aromatic systems in compounds **1** and **4** offer an extended conjugation and, thus, are presumed to experience similar electronic properties and behavior despite having methoxy and hydroxy substituents, respectively.

#### 3.6.1. Frontier Molecular Orbital Analysis and Electrostatic Potential Map

**Figure 15:**
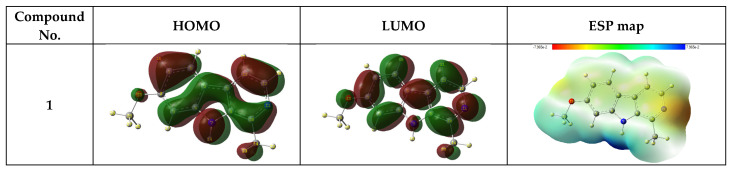

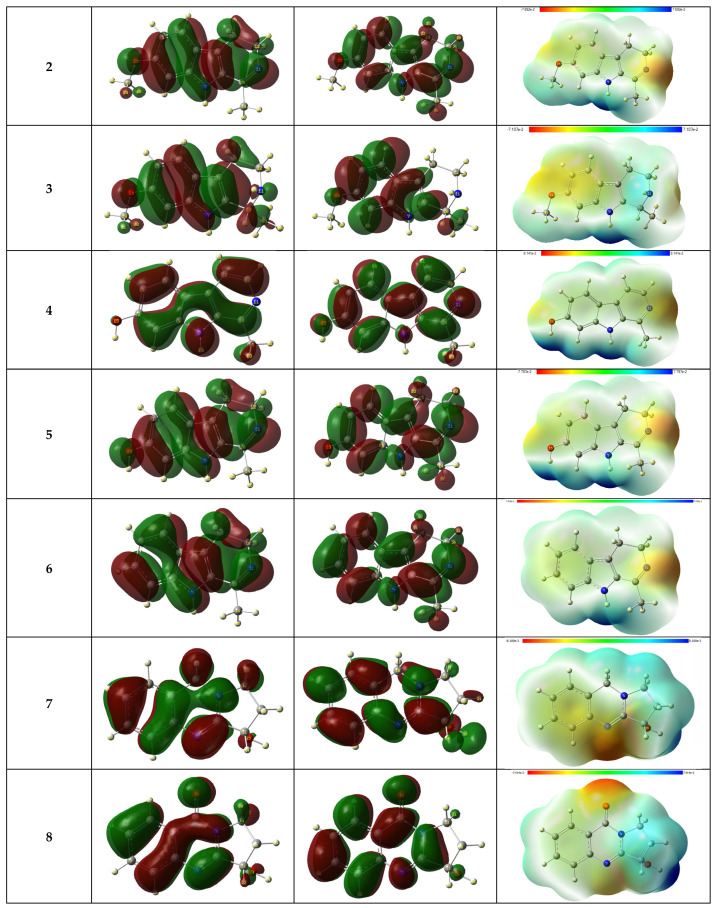
Frontier molecular orbitals (HOMO and LUMO) and electrostatic potential (ESP) maps of the stable conformers for the neutral form of compounds **1**–**8** by B3LYP method with 6-311G++(d,p) basis set using water as the solvent [49].

The analysis of the frontier molecular orbitals (FMOs) comprising the highest occupied molecular orbitals (HOMOs) and lowest unoccupied molecular orbitals (LUMOs) is fundamental to understanding the chemical reactivity, stability, identification of active sites, and hardness and softness-related features of any molecule [62,63,64,65,66]. The energy difference between these orbitals is referred to as the energy gap or band gap, and it is strongly affected by the substitution pattern present in a molecule. The energy of HOMO (E_HOMO_) indicates the electron donation capability of a molecule. Hence, high values of E_HOMO_ show the molecule’s tendency to donate electrons to electrophilic species, in addition to its correlation with the ionization potential of a molecule. The energy of LUMO (E_LUMO_) represents the ability to gain electrons, which helps to determine the electrophilic regions in a molecule and corresponds with its electron affinity. The calculated values of E_HOMO_ and E_LUMO_ for the compounds **1**–**8** present in the extract of the Harmal plant in the aqueous phase are shown in Figure 15. As expected, the shapes of the frontier molecular orbitals are similar for compounds **1** and **4** due to the presence of identical structural features. A similar trend can be observed for the pair of compounds **2** and **5**. However, it appears to be a little different for compounds **7** and **8,** and it is believed to be due to the presence of the carbonyl group in the indole system in compound **8**. The latter compounds appear to be structurally different compared to the rest of the alkaloids present in Harmal extract, as they contain benzylic rings adjacent to the indole system, which may greatly impact their electronic properties. Moreover, harmane (**6**) can be compared with tetrahydroharmine (**3**) in terms of the effect of methoxy substituent on the inhibition efficiency of both molecules. In addition to FMOs, we have also probed the molecular electrostatic potential (ESP) for all the investigated compounds [67,68]. This helps to visualize a three-dimensional distribution of the electronic charge within a molecule. The blue-colored region has the highest electrostatic potential energy and represents the electron-deficient site, while the red-colored zone indicates the lowest potential and is an indication of the electron-affluent part of the molecule. As shown in Figure 15, the nitrogen atoms in the six-membered ring attached to the indole skeleton contain the highest electronic density due to the lone pair. At the same time, the resonance phenomenon renders the nitrogen atoms of the indole skeleton to be the most electron-deficient regions in the molecules.

#### 3.6.2. E_HOMO_ and E_LUMO_ and ΔE

**Figure 16:**
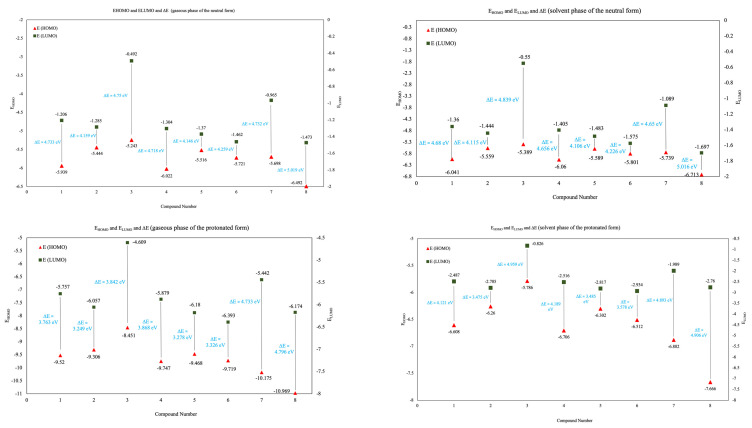
Comparative representation of E_HOMO_ and E_LUMO_ for the neutral and protonated forms of studied compounds **1**–**8** calculated in the gaseous and solvent phases using water and by applying PCM model at the B3LYP/6-311G++(d,p).

The values of FMOs are calculated and depicted in Figure 16. The order of inhibition efficiency varies according to the changing values of FMOs. Compound **3** has the largest E_HOMO_ in both states, while compound **8** has the lowest E_LUMO_ in both states. Subsequently, the energy gap values (ΔE) followed a descending order commencing with compound **8** with a value of 5.019 eV in a gaseous phase, which also remains in the same range throughout all the phases in both neutral and protonated forms. Surprisingly, ΔE starts decreasing in the following order **8** > **3** > **1** > **7** > **4** > **6** > **2** > **5**. As illustrated in Figure 16, compound **5** shows the lowest energy gap in both the gaseous and solvent states. Accordingly, it is expected to show high inhibition potency in comparison with other compounds. Similarly, compound **2** shows a similar behavior having ΔE of 3.475 eV, which is relatively close to compound **5**. Compounds **1**, **4**, **6**, and **7** have greater ΔE, and this is assumed to be related to the extended conjugation in compounds **1** and **4** and the presence of two electron-rich atoms in compounds that can give relative stability to the compounds and lower their reactivity accordingly. Compound **3** has a high energy gap, although its only difference in the conformation is the absence of the double bond between C_11_ and C_10,_ which implies its impact on the reactivity. The largest ΔE value was noted for compound **8,** which can be referred to as the presence of the carbonyl and the methoxy groups in the compound that can enhance the energy difference between both FMOs, leading to low inhibition efficiency. In the protonated form (gaseous phase), compound **3** still has the largest E_HOMO,_ and it starts to show a significant decrease for other compounds. As previously mentioned, compounds **1** and **4** have structural similarities (extended conjugation), which is why they both show relatively close values. On the other hand, the high E_HOMO_ shown by compound **2** in comparison to **5** can be influenced by the presence of methoxy/hydroxyl groups. Compound **8** has the lowest E_LUMO_ in this gaseous phase, and the values start increasing in the following order **2** < **4** < **1** < **3**. High E_LUMO_ implies a high tendency to accept electrons. E_HOMO_, E_LUMO_, and ΔE of the solvent state showed a slight elevation than the gaseous state. Compared to the neutral form, the same results are given by the same compounds (**8** and **5**) in the protonated form in terms of E_HOMO_ and E_LUMO_. However, the lowest ΔE here is calculated for compound **2** in both gaseous and aqueous phases. Further study is underway in our group to calculate the quantum chemical parameters for the major alkaloids present in the harmal extract by applying various available methods, including electron propagator theory (EPT) [69], and will be disseminated in due course.

## 4. Conclusions

This study revealed that the three types of Harmal extracts roots, leaves, and flowers (HRE, HLE, HFE) acted as efficient green corrosion inhibitors on carbon steel in 0.25 M H_2_SO_4_ media, and HRE extract showed the highest inhibitory activity amongst the three extracts studied. The % corrosion inhibition efficiency *%E* calculated from impedance and potentiodynamic polarization is in the order HRE = HLE > HFE. In contrast, the corrosion rate follows the order HRE < HLE < HFE. Moreover, the high negative values of Δ*G*_ads_ calculated from the Langmuir isotherm revealed that corrosion inhibition occurred due to the adsorption of the extract on C-steel through the chemisorption mechanism. In addition, DFT calculations were performed to visualize the frontier molecular orbitals and the energy gap between the HOMO and LUMO for the major alkaloids present in the harmal extract.

## Figures and Tables

**Table 1:** Impedance parameters for the Harmal roots extract on the C-steel in 0.25 M H_2_SO_4_ medium by fitting the equivalent circuit model 1.

Harmal Roots	*R*_s_	*R*_f_	*n*_f_	*Z*_CPEf_	*R*_ct_	*n*	*Z*_CPE_	*C*_dl_	*R*_f_ + *R*_ct_	*θ*	*IE%*
Model 1	Ω cm^2^	Ω cm^2^		µΩ^−1^s ^n^ cm^−2^	Ω cm^2^		µΩ^−1^s ^n^ cm^−2^	μF/cm^2^	Ω cm^2^		
0 ppm	6.63	9.23	0.659	2936.14	9.94	0.976	4.30 × 10^2^	375.66	19.17	0.000	0.00
20.8 ppm	6.68	64.25	0.924	131.37	15.53	0.614	8.76 × 10^2^	58.62	79.78	0.760	75.97
29.1 ppm	5.27	55.85	0.923	171.53	71.03	0.738	1.87 × 10^2^	40.25	126.88	0.849	84.89
41.5 ppm	4.94	85.11	0.937	131.41	123.01	0.761	1.37 × 10^2^	37.78	208.12	0.908	90.79
62.1 ppm	5.87	222.14	0.878	67.83	17.37	0.589	1.65 × 10^3^	138.10	239.52	0.920	92.00
82.6 ppm	7.37	22.48	0.800	62.44	319.37	0.840	4.42 × 10^1^	19.29	341.84	0.944	94.39

**Table 2:** Impedance parameters for the Harmal roots extract on the C-steel in 0.25 M H_2_SO_4_ medium by fitting the equivalent circuit model 2.

Harmal Roots	*R*_s_	*R*_f_	*n*_f_	*Z*_CPEf_	*R*_ct_	*C*_dl_	*R*_f_ + *R*_ct_	*θ*	*IE%*	*W*
Model 2	Ω cm^2^	Ω cm^2^		µΩ^−1^s ^n^ cm^−2^	Ω cm^2^	μF/cm^2^	Ω cm^2^			Ss^1/2^
0 ppm	6.58	15.11	0.902	430.08	4.12	30475.43	19.23	0.000	0.00	0.00873
20.8 ppm	6.42	28.59	1.000	305.35	50.92	91.25	79.44	0.758	75.79	0.00127
29.1 ppm	5.17	118.79	0.824	105.79	8.46	82.38	127.25	0.849	84.89	0.00296
41.5 ppm	4.80	119.49	0.815	95.86	87.22	128.09	206.71	0.907	90.70	0.00325
62.1 ppm	5.73	210.78	0.900	70.06	27.425	99.36	238.20	0.919	91.93	0.00178
82.6 ppm	4.91	131.96	0.88	44.86	176.15	57.11	308.10	0.938	93.76	0.00254

**Table 3:** Impedance parameters for the Harmal leaf extract on the C-steel in 0.25 M H_2_SO_4_ medium by fitting the equivalent circuit model 1.

Harmal Leaves	*R*_s_	*R*_f_	*n*_f_	*Z*_CPEf_	*R*_ct_	*n*	*Z*_CPE_	*C*_dl_	*R*_f_ + *R*_ct_	*θ*	*IE%*
Model 1	Ω cm^2^	Ω cm^2^		µΩ^−1^s ^n^ cm^−2^	Ω cm^2^		µΩ^−1^s ^n^ cm^−2^	μF/cm^2^	Ω cm^2^		
0 ppm	6.6	9.23	0.6588	2936.14	9.94	0.976	4.30 × 10^2^	375.66	19.17	0.000	0.00
20.8 ppm	6.2	22.64	0.8894	265.76	2.45	0.613	3.18 × 10^3^	147.48	25.10	0.236	23.61
29.1 ppm	6.0	66.26	0.8968	123.87	7.48	0.632	1.55 × 10^3^	115.54	73.73	0.740	74.00
41.5 ppm	5.9	65.65	0.6606	99.52	147.84	0.698	1.87 × 10^2^	39.54	213.50	0.910	91.02
62.1 ppm	5.5	164.28	0.9515	53.01	17.23	0.666	8.83 × 10^2^	107.89	181.51	0.894	89.44
82.6 ppm	4.5	333.09	0.8875	47.05	0.90	0.265	5.49 × 10^4^	12.80	333.99	0.943	94.26

**Table 4:** Impedance parameters for the Harmal leaf extract on the C-steel in 0.25 M H_2_SO_4_ medium by fitting the equivalent circuit model 2.

Harmal Leaves	*R*_s_	*R*_f_	*n*_f_	*Z*_CPEf_	*R*_ct_	*C*_dl_	*R*_f_ + *R*_ct_	*θ*	*IE%*	*W*
Model 2	Ω cm^2^	Ω cm^2^		µΩ^−1^s ^n^ cm^−2^	Ω cm^2^	μF/cm^2^	Ω cm^2^			Ss^1/2^
0 ppm	6.6	15.11	0.9024	430.08	4.12	30,475.43	19.23	0.000	0.00	8.73 × 10^−3^
20.8 ppm	6.1	2.10	1.0000	559.18	22.92	185.20	25.01	0.231	23.11	3.95 × 10^−3^
29.1 ppm	5.8	41.16	0.9701	196.00	32.13	84.76	73.29	0.738	73.76	2.27 × 10^−3^
41.5 ppm	6.3	45.08	0.9751	151.38	39.64	43.86	84.72	0.773	77.30	1.10 × 10^−3^
62.1 ppm	5.3	132.16	1.0000	63.88	48.51	62.88	180.67	0.894	89.36	2.06 × 10^−3^
82.6 ppm	4.7	143.77	0.8924	68.09	186.15	71.61	329.92	0.942	94.17	5.10 × 10^−3^

**Table 5:** Impedance parameters for the Harmal flower extract on the C-steel in 0.25 M H_2_SO_4_ medium by fitting the equivalent circuit model 1.

Harmal Flowers	*R*_s_	*R*_f_	*n*_f_	*Z*_CPEf_	*R*_ct_	*n*	*Z*_CPE_	*C*_dl_	*R*_f_ + *R*_ct_	*θ*	*IE%*
Model 1	Ω cm^2^	Ω cm^2^		µΩ^−1^s ^n^ cm^−2^	Ω cm^2^		µΩ^−1^s ^n^ cm^−2^	μF/cm^2^	Ω cm^2^		
0 ppm	6.63	9.23	0.659	2936.14	9.94	0.976	4.30 × 10^2^	375.66	19.17	0.000	−0.01
20.8 ppm	6.38	32.73	0.937	238.71	20.29	0.734	5.72 × 10^2^	114.15	53.02	0.638	63.85
29.1 ppm	6.29	55.65	0.895	154.82	2.99	0.538	4.73 × 10^3^	121.90	58.64	0.673	67.31
41.5 ppm	4.59	102.30	0.876	140.74	4.04	0.542	4.56 × 10^3^	155.72	106.34	0.820	81.97
62.1 ppm	6.09	126.38	0.832	86.06	2.50	0.586	2.05 × 10^3^	49.32	128.88	0.851	85.13
82.6 ppm	4.90	3.89	0.540	4887.61	166.59	0.87	6.94 × 10^1^	1.56	170.49	0.888	88.76

**Table 6:** Impedance parameters for the Harmal flower extract on the C-steel in 0.25 M H_2_SO_4_ medium by fitting the equivalent circuit model 2.

Harmal Flowers	*R*_s_	*R*_f_	*n*_f_	*Z*_CPEf_	*R*_ct_	*C*_dl_	*R*_f_ + *R*_ct_	*θ*	*IE%*	*W*
Model 2	Ω cm^2^	Ω cm^2^		µΩ^−1^s ^n^ cm^−2^	Ω cm^2^	μF/cm^2^	Ω cm^2^			Ss^1/2^
0 ppm	6.58	15.11	0.902	430.08	4.12	30,475.43	19.23	0.000	0.00	8.73 × 10^−3^
20.8 ppm	6.23	39.07	0.924	229.16	13.83	127.97	52.89	0.636	63.64	3.61 × 10^−3^
29.1 ppm	6.25	15.20	0.954	277.10	43.04	163.82	58.23	0.670	66.98	3.01 × 10^−3^
41.5 ppm	4.54	43.97	0.884	239.31	61.53	190.31	105.50	0.818	81.77	2.98 × 10^−3^
62.1 ppm	5.91	84.91	0.918	115.56	42.77	49.33	127.68	0.849	84.94	1.49 × 10^−3^
82.6 ppm	4.82	75.36	0.87	99.82	93.55	97.23	168.91	0.886	88.62	2.77 × 10^−3^

**Table 7:** Phase angles and alpha values (slopes) from the Bode phase and Bode plots for different types of Harmal extract at various concentrations.

				Harmal Extract			
		0 ppm	20.8 ppm	29.1 ppm	41.5 ppm	62.1 ppm	82.6 ppm
Harmal Roots	Phase angle °	−27.87	−41.30	−49.06	−56.00	−57.16	−62.52
	Frequency Hz	79.00	125.60	252.40	252.40	158.40	315.50
	Slope α	−0.3635	−0.4804	−0.5491	−0.6417	−0.6619	−0.7190
	*R^2^*	0.9646	0.9955	0.9973	0.9982	0.9999	0.9968
Harmal Flowers	Phase angle °	−27.87	−40.23	−44.08	−53.23	−50.23	−57.58
	Frequency Hz	75.00	125.60	125.60	125.60	252.40	198.60
	Slope α	−0.3635	−0.4945	−0.5492	−0.6324	−0.5995	−0.6571
	*R^2^*	0.9492	0.9875	0.9776	0.9886	0.9652	0.9964
Harmal Leaves	Phase angle °	−27.87	−31.78	−45.74	−41.88	−59.68	−66.28
	Frequency Hz	79.00	125.60	125.60	252.40	158.40	198.60
	Slope α	−0.3635	−0.3995	−0.5557	−0.4851	−0.7075	−0.7671
	*R^2^*	0.9892	0.9958	0.9969	0.9930	0.9982	0.9984

**Table 8:** Polarization parameters for various concentrations of Harmal roots extract on the C-steel in 0.25 M H_2_SO_4_ medium.

Harmal Roots	*I*_corr_ μA	*E*_corr_ mV	*β*a V/Decade	*β*c V/Decade	Corrosion Rate mpy	*χ* ^2^	*I*_corr_ μA/cm^2^	*θ*	*IE%*
0 ppm	1710.0	−397.0	0.4128	0.4970	1554.00	6.532	3401.63	0.000	0.0
20.8 ppm	585.0	−444.0	0.2681	0.3553	532.00	21.51	1163.72	0.658	65.8
29.1 ppm	206.6	−417.0	0.2066	0.3011	187.40	33.64	410.98	0.879	87.9
41.5 ppm	102.2	−432.0	0.1808	0.2369	92.47	40.54	203.30	0.940	94.0
62.1 ppm	102.0	−443.0	0.1886	0.2333	92.73	38.78	202.90	0.940	94.0
82.6 ppm	69.7	−446.0	0.1687	0.2244	63.34	43.41	138.65	0.959	95.9

**Table 9:** Polarization parameters for various concentrations of Harmal leaf extract on the C-steel in 0.25 M H_2_SO_4_ medium.

Harmal Leaves	*I*_corr_ μA	*E*_corr_ mV	*β*a V/Decade	*β*c V/Decade	Corrosion Rate mpy	*χ* ^2^	*I*_corr_ μA/cm^2^	*θ*	*IE%*
0 ppm	1710.0	−397.0	0.4128	0.4970	1554.0	6.532	3401.63	0.000	0.0
20.8 ppm	1550.0	−457.0	0.3539	0.4447	1412.0	11.01	3083.35	0.094	9.4
29.1 ppm	567.0	−449.0	0.2669	0.3593	515.7	22.63	1127.91	0.668	66.8
41.5 ppm	338.0	−462.0	0.2423	0.3060	307.2	30.61	672.37	0.802	80.2
62.1 ppm	180.0	−436.0	0.2186	0.2565	163.7	38.45	358.07	0.895	89.5
82.6 ppm	94.7	−452.0	0.1823	0.2136	86.1	51.29	188.38	0.945	94.5

**Table 10:** Polarization parameters for various concentrations of Harmal flower extract on the C-steel in 0.25 M H_2_SO_4_ medium.

Harmal Flowers	*I*_corr_ μA	*E*_corr_ mV	*β*a V/Decade	*β*c V/Decade	Corrosion Rate mpy	*χ* ^2^	*I*_corr_ μA/cm^2^	*θ*	*IE%*
0 ppm	1710.0	−397.0	0.4128	0.4970	1554.00	6.532	3401.63	0.000	0.0
20.8 ppm	767.0	−451.0	0.2897	0.3984	696.90	16.57	1525.76	0.551	55.1
29.1 ppm	653.0	−444.0	0.2684	0.3540	593.80	23.17	1298.99	0.618	61.8
41.5 ppm	344.0	−425.0	0.2239	0.2850	313.00	33.80	684.30	0.799	79.9
62.1 ppm	261.0	−464.0	0.2281	0.2783	237.10	28.18	519.20	0.847	84.7
82.6 ppm	199.0	−460.0	0.2007	0.2584	180.70	37.51	395.86	0.884	88.4

**Table 11:** Percent inhibition efficiency (IE%) toward steel corrosion in sulfuric acid by Harmal roots, leaves, and flower extracts compared with other reported systems at various concentrations (ppm) [58,59,60,61].

Harmal (ppm) This Work	HRE IE% EIS 0.25 M H_2_SO_4_ C-Steel	HLE IE% EIS 0.25 M H_2_SO_4_ C-Steel	HFE IE% EIS 0.25 M H_2_SO_4_ C-Steel	Date Palm Seed (ppm)	IE% WLS 0.5 M HCl C-Steel	Tea Tree (ppm)	IE% PDP 1 M HCl Mild Steel	Ginkgo Leaf Extract (ppm)	IE% PDP 1 M HCl X-70 Steel	Brassica Oleracea L (ppm)	IE% PDP 0.50 M H_2_SO_4_ Q235 Steel
20.8	65.8	9.4	55.1	800	71	150	56.5	25	62.0	50	81.2
29.1	87.9	66.8	61.8	900	82	300	64.0	50	70.8	100	86.0
41.5	94.0	80.2	79.9	1200	88	750	75.6	100	86.3	200	90.0
62.1	94.0	89.5	84.7	1400	95	2250	78.6	200	90.0	300	92.7
82.6	95.9	94.5	88.4	2000	91						
This work				[58]		[59]		[60]		[61]	

## Data Availability

All data are available in the main text or the Electronic Supplementary Information (ESI).

## Supplementary Materials

The following are available online at https://www.mdpi.com/article/10.3390/molecules27217250/s1, Figure S1: The open circuit potential E_OCP_ plot versus time (s) of C-steel with and without the addition of different concentrations of Harmal extracts (a) Roots, (b) Leaves and (c) Flowers in 0.25M H_2_SO_4_, Table S1: Computed energies for the compounds 1-8 present in the Harmal extract calculated at B3LYP/6-311G++(d,p) [values are in Hartree], Table S2: Cartesian coordinates for the compounds 1-8 present in the Harmal extract computed at B3LYP/6-311G++(d,p).

## Sample Availability

The extracted samples of the Harmal plant are available from the authors.
