# Supplementary material for: An Integrated Experimental and Theoretical Studies on the Corrosion Inhibition of Carbon Steel by Harmal Extracts
Source: Molecules. 2022 Oct 25;27(21):7250. doi: 10.3390/molecules27217250 (PMC9657846; doi:10.3390/molecules27217250)
Supplement: Supplementary file 1 [file molecules-27-07250-s001.zip › molecules-1939610-supplementary.pdf]

Electronic Supplementary Information for the DFT Studies for manuscript:

An Integrated Experimental and Theoretical Studies on the Corrosion Inhibition of Carbon Steel by Harmal Extracts

Hassan H. Hammud,[*a] Sarah A. Maache,[*b] Nasreen Al Otaibi,[*a] Nadeem S. Sheikh[*c]

[a] Department of Chemistry, College of Science, King Faisal University, P.O. Box 400, Al-Ahsa 31982, Saudi Arabia

[b] Leading National Academy, Khobar Niagara College, Saudi Arabia

[c] Chemical Sciences, Faculty of Science, Universiti Brunei Darussalam, Jalan Tungku Link, Gadong BE1410, Brunei Darussalam

[*] Correspondence: hhammoud@kfu.edu.sa (H.H.H.); sara.maache@lna.edu.sa (S.A.M.); nbalotaibi@kfu.edu.sa (N.A.O); nadeem.sheikh@ubd.edu.bn (N.S.S.)

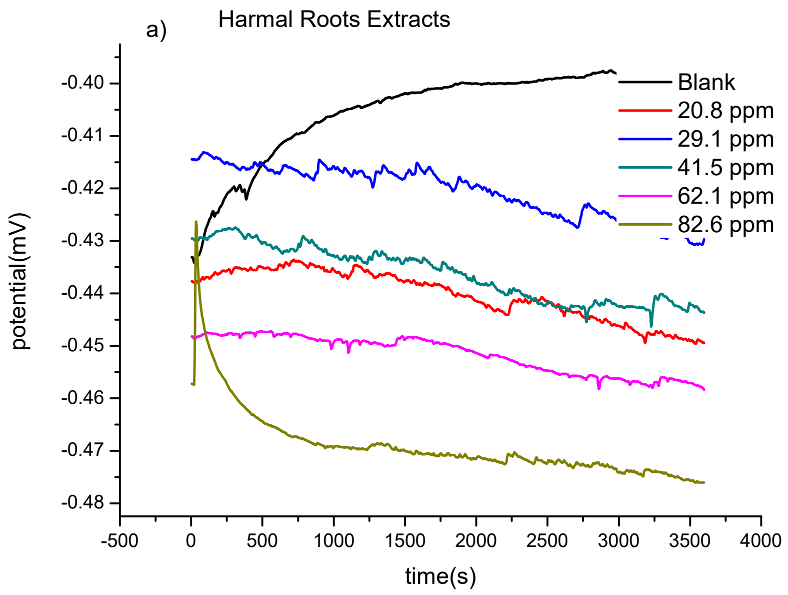

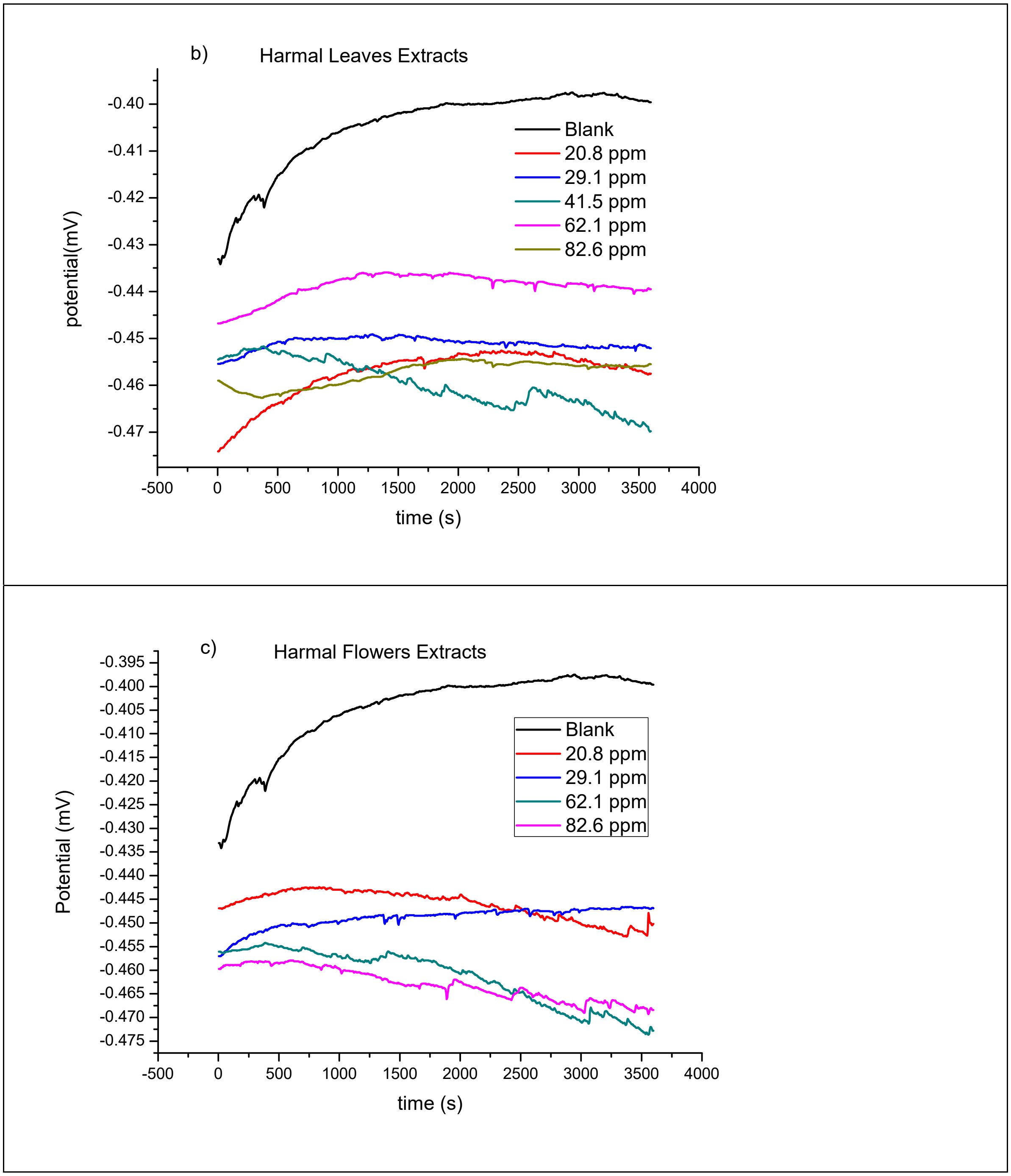

Figure S1: The open circuit potential E_{OCP} plot versus time (s) of C-steel with and without the addition of different concentrations of Harmal extracts a) Roots, b) Leaves and c) Flowers in 0.25M H_2SO_4

Table S1: Computed energies for the compounds **1-8** present in the Harmal extract calculated at B3LYP/6-311G++(d,p) [values are in Hartree]

Compound No.	Species (Phase)	Total Electronic Energy	Sum of Electronic and Zero-point Energies	Sum of Electronic and Thermal Enthalpies	Gibbs Free Energy
**1**	Neutral (gas)	-687.5277901	-687.304343	-687.290022	-687.344143
	Neutral (aq.)	-687.5406854	-687.317247	-687.302999	-687.356944
	Protonated (gas)	-687.9251521	-687.687517	-687.673067	-687.727533
	Protonated (aq.)	-687.9971114	-687.759501	-687.745080	-687.799646
**2**	Neutral (gas)	-688.7117287	-688.465421	-688.450556	-688.505605
	Neutral (aq.)	-688.7243585	-688.478139	-688.463324	-688.518279
	Protonated (gas)	-689.1191448	-688.858815	-688.843665	-688.899458
	Protonated (aq.)	-689.1894665	-688.928885	-688.914004	-688.968883
**3**	Neutral (gas)	-689.9216611	-689.651129	-689.635937	-689.691722
	Neutral (aq.)	-689.9337581	-689.663108	-689.648046	-689.703495
	Protonated (gas)	-690.3010979	-690.015563	-690.000263	-690.056132
	Protonated (aq.)	-690.3886176	-690.102361	-690.087235	-690.142728
**4**	Neutral (gas)	-648.2183518	-648.023209	-648.010222	-648.061096
	Neutral (aq.)	-648.2332676	-648.038105	-648.025178	-648.076147
	Protonated (gas)	-649.8069348	-649.574731	-649.561076	-649.613343
	Protonated (aq.)	-648.6893472	-648.479642	-648.466663	-648.518024
**5**	Neutral (gas)	-649.4022675	-649.184287	-649.170751	-649.222506
	Neutral (aq.)	-649.4169048	-649.198928	-649.185482	-649.237061
	Protonated (gas)	-649.8069348	-649.574731	-649.561076	-649.613343
	Protonated (aq.)	-649.88145	-649.649333	-649.635726	-649.687704
**6**	Neutral (gas)	-574.1563024	-573.942123	-573.929962	-573.978773
	Neutral (aq.)	-574.1665805	-573.952505	-573.940363	-573.989180
	Protonated (gas)	-574.5560344	-574.327867	-574.315460	-574.364975
	Protonated (aq.)	-574.628648	-574.400306	-574.388002	-574.437187
**7**	Neutral (gas)	-611.2871074	-611.072172	-611.060255	-611.109210
	Neutral (aq.)	-611.2959079	-611.081551	-611.069390	-611.118909
	Protonated (gas)	-611.6806331	-611.452279	-611.439805	-611.490572
	Protonated (aq.)	-611.7546719	-611.526199	-611.513717	-611.564209
**8**	Neutral (gas)	-685.3563145	-685.160173	-685.147481	-685.198362
	Neutral (aq.)	-685.3730315	-685.176695	-685.164226	-685.214318
	Protonated (gas)	-685.7303235	-685.520642	-685.507837	-685.558974
	Protonated (aq.)	-685.8106458	-685.600662	-685.587881	-685.638723

Table S2: Cartesian coordinates for the compounds **1-8** present in the Harmal extract computed at B3LYP/6-311G++(d,p)

Compound No. **1** [Neutral (Gas)]

C	2.49366600	-1.57868800	0.00007400
C	2.90185400	-0.22591800	0.00013500
C	1.97027700	0.81036000	0.00010300
C	0.61747100	0.45275200	0.00002100
C	0.18672300	-0.89508300	-0.00003100
C	1.15265600	-1.91139800	-0.00000800
N	-0.49670900	1.27336800	-0.00004000
C	-1.64426100	0.48804600	-0.00009600
C	-1.25868200	-0.87129700	-0.00009100
C	-2.99246700	0.86328200	-0.00015700
N	-3.93964100	-0.07734200	-0.00020000
C	-3.58493900	-1.37641900	-0.00018900
C	-2.27372300	-1.83286700	-0.00014600
C	-3.41512000	2.30793200	-0.00012500
O	4.25246700	-0.03543200	0.00019500
H	3.26334600	-2.34005400	0.00009800
H	2.26920500	1.84972700	0.00012900
H	0.85542600	-2.95405100	-0.00004500
H	-0.47858800	2.27901400	0.00006700
H	-4.40793100	-2.08453500	-0.00024100
H	-2.05993200	-2.89520000	-0.00015500
H	-4.50300600	2.36462600	-0.00052900
H	-3.04060100	2.83597800	0.88450100
H	-3.03991200	2.83623300	-0.88430400
C	4.75600700	1.29260400	0.00039000
H	4.43971100	1.84004800	-0.89443900
H	4.43948900	1.83986300	0.89525200
H	5.84073000	1.19978800	0.00051100

Compound No. **1** [Neutral (Aq.)]

C	2.49036100	-1.58342600	0.00007200
C	2.89902100	-0.22800600	0.00010300
C	1.96884400	0.80913700	0.00008100
C	0.61572900	0.45069400	0.00002100
C	0.18466600	-0.89936100	-0.00001800
C	1.14919500	-1.91857500	0.00000900
N	-0.49533800	1.27226600	-0.00001700
C	-1.64079800	0.49062400	-0.00007000
C	-1.25913300	-0.87245100	-0.00008600
C	-2.98962800	0.86945400	-0.00012900
N	-3.93842700	-0.07467400	-0.00019600
C	-3.58466800	-1.37816700	-0.00020900
C	-2.27454700	-1.83509100	-0.00015100
C	-3.40543100	2.31504500	-0.00012500
O	4.24893800	-0.03712100	0.00016600
H	3.25707700	-2.34843600	0.00009900
H	2.26496300	1.84872700	0.00010500
H	0.85289000	-2.96142100	-0.00001800
H	-0.47017900	2.27992000	0.00009900
H	-4.40654900	-2.08756200	-0.00025300
H	-2.06151700	-2.89734400	-0.00015900
H	-4.49277600	2.38626900	-0.00059600
H	-3.02310300	2.83991800	0.88175000
H	-3.02231000	2.84017400	-0.88150100
C	4.75344100	1.30021900	0.00038600
H	4.43333100	1.84245000	-0.89413400
H	4.43307100	1.84225200	0.89493200
H	5.83764500	1.20830700	0.00053000

Compound No. **1** [Protonated (Gas)]

C	-2.51593200	1.57722400	-0.00009900
C	-2.93031300	0.21358500	-0.00010400
C	-1.99996600	-0.82895500	-0.00013500
C	-0.65495700	-0.46710900	-0.00011500
C	-0.22047200	0.88458700	-0.00010000
C	-1.18734000	1.91390600	-0.00009600
N	0.46167500	-1.29069200	-0.00016000
C	1.59764400	-0.51330600	-0.00007500
C	1.20162000	0.86407600	-0.00004200
C	2.92465200	-0.89637200	0.00002000
N	3.83070500	0.12078200	0.00018400
C	3.51365100	1.44805900	0.00021900
C	2.20298300	1.85011800	0.00007700
C	3.41209200	-2.31150700	-0.00007400
O	-4.25843200	0.03954500	-0.00013700
H	-3.29042500	2.33320900	-0.00007800
H	-2.30337600	-1.86629400	-0.00026200
H	-0.89004300	2.95567600	-0.00006100
H	0.43529900	-2.29809200	-0.00020100
H	4.34904100	2.13346400	0.00029200
H	1.96911100	2.90602500	0.00007200
H	4.50205400	-2.36317700	0.00037200
H	3.05380500	-2.84560000	0.88470200
H	3.05451700	-2.84527300	-0.88533900
H	4.81179700	-0.12977600	0.00019500
C	-4.80658400	-1.28380400	0.00044000
H	-4.50458200	-1.82976900	-0.89758100
H	-4.50347800	-1.82947500	0.89825700
H	-5.88539700	-1.15091800	0.00106700

Compound No. **1** [Protonated (Aq.)]

C	-2.51731200	1.58170500	-0.00005900
C	-2.92911600	0.22102000	-0.00009900
C	-2.00316200	-0.81968500	-0.00025700
C	-0.65291900	-0.46041100	-0.00019700
C	-0.22012400	0.89058700	-0.00011300
C	-1.18345400	1.91815400	-0.00009500
N	0.45603700	-1.28430800	-0.00023500
C	1.59284700	-0.50881800	-0.00009600
C	1.20920600	0.86589200	-0.00003900
C	2.92289900	-0.89946800	0.00001000
N	3.82675600	0.11098700	0.00020300
C	3.51959400	1.43777700	0.00022200
C	2.21037100	1.84835800	0.00006100
C	3.40066300	-2.31498500	-0.00001000
O	-4.27032600	0.03900100	-0.00012200
H	-3.28643200	2.34377700	0.00009200
H	-2.30038400	-1.85835900	-0.00042300
H	-0.88405500	2.95937900	-0.00004800
H	0.42843800	-2.29294700	-0.00011900
H	4.36017600	2.11511100	0.00032700
H	1.98112000	2.90477100	0.00007800
H	4.48938100	-2.36705000	0.00026200
H	3.03233300	-2.84291600	0.88329800
H	3.03276600	-2.84275000	-0.88359800
H	4.80914800	-0.14061900	0.00031200
C	-4.79165300	-1.29578600	0.00050800
H	-5.87372900	-1.18773300	0.00116700
H	-4.47694500	-1.83791700	-0.89492100
H	-4.47579900	-1.83754800	0.89574400

Compound No. **2** [Neutral (Gas)]

C	-2.52357300	-1.57677700	-0.11921700
C	-2.95381400	-0.23176400	-0.01181800
C	-2.04082400	0.81404600	0.06655700
C	-0.68058700	0.47769800	0.03142000
C	-0.22332400	-0.86557600	-0.06449800
C	-1.18179500	-1.89421200	-0.14462400
N	0.42559700	1.29586600	0.07691500
C	1.57110800	0.50384700	0.04439900
C	1.20577100	-0.81826800	-0.04440500
C	2.96136300	0.93771500	-0.02840600
N	3.92361300	0.09584200	0.09372900
C	3.58197600	-1.29676800	0.41486900
C	2.28300900	-1.84628000	-0.20695300
C	3.27680500	2.39135400	-0.27264800
O	-4.31055500	-0.06716100	0.00597500
H	-3.28441600	-2.34520000	-0.17765600
H	-2.35549700	1.84602900	0.14470900
H	-0.87209700	-2.93038100	-0.22301300
H	0.40685800	2.29389500	0.20281900
H	4.43224700	-1.91978200	0.12913600
H	3.50230400	-1.35564400	1.51053200
H	2.42929800	-2.07003600	-1.27340600
H	2.02638700	-2.79310800	0.27761600
H	4.35666200	2.52155300	-0.32892600
H	2.82300600	2.74484800	-1.20446800
H	2.89291400	3.01823900	0.54103400
C	-4.83418400	1.24677600	0.12045300
H	-4.51787900	1.72324700	1.05534000
H	-4.53384800	1.87293600	-0.72743500
H	-5.91757300	1.13798800	0.12064100

Compound No. **2** [Neutral (Aq.)]

C	-2.51892400	-1.58222200	-0.12414800
C	-2.95045400	-0.23531400	-0.01249900
C	-2.03941200	0.81124000	0.07089200
C	-0.67780600	0.47538600	0.03582400
C	-0.21908100	-0.86969000	-0.06424600
C	-1.17624000	-1.90073700	-0.14879800
N	0.42382300	1.29479900	0.08604100
C	1.56929900	0.50590100	0.05119800
C	1.20918100	-0.81987400	-0.04303900
C	2.95692600	0.94428000	-0.02970000
N	3.92246300	0.09935100	0.08844900
C	3.58002400	-1.29487300	0.42082800
C	2.28755200	-1.84604900	-0.20756400
C	3.26040900	2.39760700	-0.28166800
O	-4.30781700	-0.07021800	0.00435800
H	-3.27636500	-2.35446400	-0.18655100
H	-2.35187500	1.84297000	0.15326200
H	-0.86640900	-2.93666600	-0.22966300
H	0.39845700	2.29514400	0.20747600
H	4.43071100	-1.92191900	0.14605300
H	3.48978500	-1.34732600	1.51589500
H	2.43797900	-2.06637900	-1.27403000
H	2.03283700	-2.79470100	0.27285300
H	4.33745000	2.54417900	-0.35497200
H	2.78703100	2.74477200	-1.20525600
H	2.87823100	3.02146100	0.53369600
C	-4.83214100	1.25295600	0.11959100
H	-4.51490900	1.72354200	1.05505300
H	-4.52537100	1.87499400	-0.72669800
H	-5.91501500	1.14542700	0.11656300

Compound No. **2** [Protonated (Gas)]

C	2.53267700	-1.58008000	0.11290900
C	2.97293100	-0.22045200	0.01129600
C	2.06882800	0.83630100	-0.06414900
C	0.71188300	0.50535000	-0.03403100
C	0.24842700	-0.84846200	0.05072300
C	1.20445600	-1.89540700	0.13402100
N	-0.38152200	1.32991400	-0.06900800
C	-1.53383600	0.54169200	-0.05910400
C	-1.15345000	-0.80844000	0.02279900
C	-2.86485400	0.96244400	0.04414900
N	-3.81067400	0.02541300	-0.02306500
C	-3.52218500	-1.36846400	-0.41201400
C	-2.20486500	-1.86104500	0.19762700
C	-3.26543600	2.39235700	0.22944900
O	4.30415700	-0.08076400	-0.00055500
H	3.29775500	-2.34375400	0.16895100
H	2.39436900	1.86469900	-0.13202900
H	0.88793600	-2.92886600	0.20720500
H	-0.34614400	2.32321500	-0.23499400
H	-4.36192800	-1.98539000	-0.09458900
H	-3.46272100	-1.40414500	-1.50636100
H	-2.32597800	-2.07899900	1.26643900
H	-1.92596600	-2.79954600	-0.28585300
H	-4.31754000	2.48323700	0.50213500
H	-2.66496500	2.86692100	1.00815700
H	-3.11182900	2.94705800	-0.70223200
H	-4.77737600	0.31110200	0.04586200
C	4.88493500	1.22508900	-0.10243600
H	4.58859200	1.70887600	-1.03709900
H	4.60073400	1.84335000	0.75345000
H	5.96011100	1.06576600	-0.09752300

Compound No. **2** [Protonated (Aq.)]

C	2.53599400	-1.58407100	0.11415800
C	2.97380200	-0.22892200	0.01215800
C	2.07280100	0.82488300	-0.06364000
C	0.71078100	0.49465300	-0.03225600
C	0.24854200	-0.85567200	0.05519500
C	1.20176000	-1.89947500	0.13596300
N	-0.37837700	1.31910700	-0.07455400
C	-1.52701100	0.53324400	-0.05634600
C	-1.16215700	-0.81128400	0.02924800
C	-2.86826400	0.96758500	0.03937700
N	-3.80690100	0.04253200	-0.04274300
C	-3.52922800	-1.35947100	-0.41219800
C	-2.21798200	-1.85885400	0.20083200
C	-3.24994300	2.39642800	0.24039600
O	4.31981700	-0.07961500	-0.00233900
H	3.29502500	-2.35445400	0.17021500
H	2.39176100	1.85456800	-0.13656000
H	0.88380200	-2.93262400	0.20857400
H	-0.34352100	2.31887400	-0.20552900
H	-4.37590600	-1.96015200	-0.08638700
H	-3.47441400	-1.40308800	-1.50547500
H	-2.34383000	-2.07723900	1.26849800
H	-1.94327900	-2.79703300	-0.28446200
H	-4.32381400	2.50674300	0.38541800
H	-2.72807000	2.81185000	1.10496200
H	-2.95831300	2.98031300	-0.63798200
H	-4.77497700	0.32882000	0.02658500
C	4.86980300	1.23897100	-0.10757100
H	4.55841700	1.71810500	-1.03946300
H	4.57277700	1.85464600	0.74552400
H	5.94937600	1.10802600	-0.10602300

Compound No. **3** [Neutral (Gas)]

C	2.56179200	1.57762100	0.15898600
C	3.02189900	0.24753100	0.04477000
C	2.13064300	-0.81225200	-0.09878500
C	0.76620600	-0.49888300	-0.12575400
C	0.27832400	0.82711500	-0.01442800
C	1.20985200	1.86840200	0.13097600
N	-0.32815600	-1.33128100	-0.25378800
C	-1.48979900	-0.56608200	-0.23272000
C	-1.15919300	0.75337000	-0.08091000
C	-2.88694100	-1.11224800	-0.35758300
N	-3.85828700	-0.01161900	-0.52788000
C	-3.57394900	1.18236100	0.28130000
C	-2.20181900	1.82838900	-0.00059100
O	4.38448000	0.10302700	0.08811300
H	3.30198200	2.36059900	0.26911000
H	2.46694000	-1.83697000	-0.18520100
H	0.87851800	2.89732800	0.22022200
H	-4.38114900	1.89993700	0.11174900
H	-3.62529700	0.89356100	1.33555600
H	-2.24193900	2.40442300	-0.93589700
H	-1.96594500	2.54833200	0.79218400
H	-0.28893800	-2.33057900	-0.36337300
H	-2.95385500	-1.72171500	-1.26811300
H	-3.88814300	0.25208000	-1.50740400
C	-3.28519400	-2.01031900	0.82655200
H	-4.28653100	-2.41411400	0.66398900
H	-2.58618700	-2.84419200	0.94316900
H	-3.28490200	-1.44593100	1.76202400
C	4.92978700	-1.20059700	-0.02028500
H	4.59724600	-1.84546600	0.80173200
H	4.66702000	-1.66820900	-0.97652400
H	6.01079700	-1.07944400	0.03437800

Compound No. **3** [Neutral (Aq.)]

C	2.56031300	1.58270700	0.16453700
C	3.02209000	0.25200800	0.04421100
C	2.13206400	-0.80909900	-0.09973700
C	0.76587000	-0.49787400	-0.12139500
C	0.27508000	0.83017000	-0.00567000
C	1.20572700	1.87313000	0.14110400
N	-0.32436700	-1.33169500	-0.24746700
C	-1.48628500	-0.56981100	-0.22589300
C	-1.16255700	0.75286900	-0.07157500
C	-2.88179700	-1.12107600	-0.35400000
N	-3.85278300	-0.02042000	-0.55196500
C	-3.58376700	1.17803600	0.26229300
C	-2.20914200	1.82559500	-0.00013500
O	4.38686900	0.10634800	0.08150200
H	3.29594300	2.37075900	0.27575100
H	2.46720200	-1.83324600	-0.19141500
H	0.87392400	2.90164800	0.23499800
H	-4.38517400	1.89706800	0.07514900
H	-3.65222800	0.89204700	1.31619100
H	-2.24008000	2.40304300	-0.93426100
H	-1.98678300	2.54468600	0.79683700
H	-0.28084200	-2.32984600	-0.38088900
H	-2.93641000	-1.74430400	-1.25410200
H	-3.84180900	0.24546100	-1.53227400
C	-3.28342800	-2.00016700	0.84246800
H	-4.27376200	-2.42872600	0.67285400
H	-2.57279600	-2.81923600	0.98193600
H	-3.30869000	-1.41775600	1.76694300
C	4.93165100	-1.20698000	-0.02898500
H	4.59714100	-1.84659200	0.79391600
H	4.66215000	-1.67017400	-0.98340300
H	6.01240300	-1.08785000	0.02242900

Compound No. **3** [Protonated (Gas)]			
C	2.58622700	1.57153900	0.16765900
C	3.05565500	0.23818100	0.04331200
C	2.16859200	-0.82612000	-0.09782500
C	0.80507500	-0.51253300	-0.11287300
C	0.31539800	0.80808100	0.00965800
C	1.24052300	1.86061400	0.15219800
N	-0.28707100	-1.35512700	-0.24205700
C	-1.44370900	-0.59463700	-0.20001700
C	-1.11781700	0.72961800	-0.04458900
C	-2.81834400	-1.16820400	-0.32207900
N	-3.73665700	0.02347500	-0.63310300
C	-3.52689100	1.24504900	0.25663000
C	-2.13728400	1.82732200	0.01791600
O	4.40255100	0.11043300	0.07468200
H	3.32666500	2.35386500	0.27595300
H	2.50924100	-1.84795200	-0.19219100
H	0.90837500	2.88798300	0.24954800
H	-0.23400900	-2.35472000	-0.35361400
H	-3.57697800	0.30441500	-1.60453300
H	-4.32692100	1.94613000	0.01649900
H	-3.65388900	0.90009900	1.28179100
H	-2.13074600	2.43270700	-0.89764500
H	-1.92712800	2.52049700	0.83820600
H	-4.70940500	-0.28476000	-0.56715300
H	-2.90111700	-1.80966200	-1.20715800
C	-3.34358600	-1.93386400	0.89441700
H	-4.36114100	-2.30329300	0.73272700
H	-2.70706900	-2.80468300	1.06046300
H	-3.30981000	-1.33211100	1.80384600
C	4.98147600	-1.18687000	-0.04534300
H	4.66887800	-1.83648800	0.77858400
H	4.72141000	-1.64781000	-1.00369100
H	6.05743900	-1.03517800	0.00264600

Compound No. **3** [Protonated (Aq.)]

C	2.59020500	1.57964200	0.17161000
C	3.05534300	0.24857100	0.04424000
C	2.16903900	-0.81434400	-0.09946400
C	0.80279700	-0.50372500	-0.11489200
C	0.31188700	0.82072600	0.00881200
C	1.23806600	1.86872900	0.15474300
N	-0.28417900	-1.34243600	-0.24306600
C	-1.44018700	-0.58251800	-0.20450600
C	-1.12424600	0.74108400	-0.04818600
C	-2.81305200	-1.16619000	-0.32507700
N	-3.75078800	0.00331700	-0.61373300
C	-3.53911300	1.22902300	0.25691000
C	-2.15716900	1.82636100	0.00727500
O	4.41629200	0.10881900	0.07558900
H	3.32540900	2.36765200	0.28306900
H	2.50504300	-1.83725700	-0.19668900
H	0.90352000	2.89529500	0.25418600
H	-0.23896300	-2.34188400	-0.36865800
H	-3.64531800	0.27330200	-1.59495200
H	-4.34200800	1.92269700	0.01485700
H	-3.65673700	0.89896200	1.28736500
H	-2.16129500	2.41516000	-0.91737200
H	-1.95306300	2.53102300	0.81837200
H	-4.71616800	-0.31916300	-0.51448100
H	-2.88437800	-1.80412800	-1.20939500
C	-3.29523200	-1.95086100	0.89612000
H	-4.30523400	-2.33659100	0.73745700
H	-2.63261600	-2.80444000	1.04671200
H	-3.27429800	-1.34598300	1.80370600
C	4.96889100	-1.20164800	-0.04953000
H	4.69415400	-1.65721500	-1.00574500
H	6.04881400	-1.07499500	-0.00524200
H	4.64419300	-1.84825200	0.77136700

Compound No. **4** [Neutral (Gas)]			
C	-3.01468900	1.14555500	0.00005300
C	-3.26530900	-0.23964500	-0.00003200
C	-2.22849200	-1.16849300	0.00000700
C	-0.92232300	-0.67353300	0.00006200
C	-0.64478100	0.71657000	0.00005600
C	-1.71387800	1.62040800	0.00007400
N	0.27589300	-1.36397600	0.00016700
C	1.32912300	-0.45474200	0.00009000
C	0.79515300	0.85295400	0.00007000
C	2.71037700	-0.67843400	0.00004500
N	3.54705000	0.36189200	-0.00015400
C	3.05050200	1.61325300	-0.00015800
C	1.69649300	1.92139200	0.00000200
C	3.29117600	-2.06706700	0.00001800
O	-4.58102000	-0.61732600	-0.00016800
H	-3.86103900	1.82054900	0.00018400
H	-2.43264500	-2.23471700	-0.00012000
H	-1.53072500	2.68894500	0.00021200
H	0.37081800	-2.36527700	-0.00033800
H	3.78966300	2.40844300	-0.00015500
H	1.36585300	2.95332600	-0.00000900
H	4.37855200	-2.00208300	0.00082900
H	2.97689800	-2.63408700	0.88397900
H	2.97816900	-2.63345500	-0.88478600
H	-4.64809400	-1.57776400	-0.00025900

Compound No. **4** [Neutral (Aq.)]			
C	-3.01237800	1.14718700	0.00005000
C	-3.26102400	-0.24123400	-0.00004500
C	-2.22533300	-1.17044000	0.00004300
C	-0.91990000	-0.67169200	0.00011900
C	-0.64345300	0.72038000	0.00012200
C	-1.71254700	1.62628500	0.00013100
N	0.27604800	-1.36240400	0.00017100
C	1.32662400	-0.45660300	0.00014200
C	0.79514800	0.85490000	0.00014600
C	2.70909200	-0.68304100	-0.00003400
N	3.54629300	0.36128800	-0.00029800
C	3.04925300	1.61690300	-0.00023000
C	1.69615000	1.92493400	0.00006000
C	3.28309900	-2.07363100	0.00001700
O	-4.57674600	-0.62020600	-0.00024500
H	-3.85732000	1.82477000	0.00012400
H	-2.42588600	-2.23600700	-0.00008800
H	-1.53062100	2.69494000	0.00028300
H	0.36468400	-2.36616100	0.00028400
H	3.78679600	2.41357300	-0.00028500
H	1.36543600	2.95665100	0.00007900
H	4.37166300	-2.02375600	-0.00144200
H	2.96225700	-2.63730800	0.88252500
H	2.95981000	-2.63837900	-0.88088900
H	-4.64762400	-1.58255300	-0.00087100

Compound No. **4** [Protonated (Gas)]

C	-3.04880000	1.16346200	0.09826600
C	-3.33559200	-0.22948600	-0.02032800
C	-2.32932600	-1.18521300	-0.10039100
C	-1.01370500	-0.72142500	-0.05603100
C	-0.69858600	0.67363200	0.04653800
C	-1.75752700	1.61337100	0.13262000
N	0.16155400	-1.42454400	-0.09172300
C	1.22231600	-0.51713400	-0.06589600
C	0.70160900	0.78281000	0.02744300
C	2.59176700	-0.79530100	0.04327500
N	3.43148500	0.23666300	-0.01168300
C	2.99877000	1.59625300	-0.38869300
C	1.63360200	1.93997400	0.21802300
C	3.13945100	-2.17575600	0.22528400
O	-4.64539300	-0.53890500	-0.04640700
H	-3.88863700	1.84383800	0.15639100
H	-2.56199600	-2.24095500	-0.18338500
H	-1.55011200	2.67312800	0.21861600
H	0.23373700	-2.41367500	-0.27066100
H	3.76708100	2.29523500	-0.06078300
H	2.94105000	1.63536500	-1.48283900
H	1.72505600	2.16006000	1.28930300
H	1.25820400	2.84751800	-0.25930300
H	4.20554400	-2.15944500	0.45430300
H	2.61981000	-2.69531300	1.03322700
H	3.00225900	-2.75586600	-0.69319600
H	-4.78681800	-1.48970900	-0.13048800
H	4.42281900	0.05510600	0.06324900

Compound No. **4** [Protonated (Aq.)]			
C	-3.03654900	1.14869600	0.00008200
C	-3.29025300	-0.24474900	-0.00008800
C	-2.26006800	-1.17945100	-0.00000100
C	-0.95692200	-0.68178200	0.00019000
C	-0.67617300	0.71049900	0.00018700
C	-1.74339000	1.62635200	0.00021700
N	0.23601800	-1.37752800	0.00038500
C	1.28001800	-0.48101400	0.00025200
C	0.74865700	0.84314800	0.00009400
C	2.64479600	-0.72380100	0.00052800
N	3.43136200	0.37967500	0.00005200
C	2.98247000	1.66480100	-0.00038000
C	1.63549100	1.92976500	-0.00024400
C	3.27658700	-2.07770500	-0.00020800
O	-4.60056200	-0.60878700	-0.00031900
H	-3.88235100	1.82460200	0.00009400
H	-2.46263800	-2.24390300	-0.00008400
H	-1.55660200	2.69339600	0.00033200
H	0.32017800	-2.38268100	-0.00109900
H	3.74487100	2.42925700	-0.00057500
H	1.29077200	2.95447800	-0.00037600
H	4.36393600	-2.00709900	0.01933100
H	2.95482700	-2.65098100	0.87312400
H	2.98530700	-2.63524200	-0.89440300
H	-4.68829100	-1.57041700	-0.00055300
H	4.43484900	0.23530900	-0.00008200
Compound No. **5** [Neutral (Gas)]			
C	-3.04060400	1.15561500	0.10030300
C	-3.31795600	-0.22217800	-0.02563500
C	-2.30107100	-1.16469700	-0.10813700
C	-0.98400300	-0.69523500	-0.05659500
C	-0.67549400	0.69060500	0.05877400
C	-1.73611700	1.61030100	0.14157600
N	0.20400200	-1.38837600	-0.10035300
C	1.25708600	-0.47656900	-0.04980100
C	0.75143200	0.79676600	0.05042400
C	2.68593900	-0.75960100	0.02951200
N	3.55222500	0.18238600	-0.07731500
C	3.06453500	1.53345900	-0.38639000
C	1.71051600	1.93281100	0.23227900
C	3.15376000	-2.17334800	0.26168600
O	-4.64440400	-0.56964800	-0.06054700
H	-3.87697300	1.84073000	0.16094300
H	-2.52471900	-2.22311400	-0.20190700
H	-1.53692300	2.67203100	0.23404400
H	0.29483300	-2.38009100	-0.24357200
H	3.84108700	2.24124900	-0.08871200
H	2.98497500	1.59500100	-1.48186300
H	1.82538100	2.15851900	1.30214500
H	1.35608000	2.85200100	-0.24369100
H	4.24111000	-2.18809100	0.32182200
H	2.73713400	-2.58243200	1.18817000
H	2.84200200	-2.82981000	-0.55944900
H	-4.73047500	-1.52444700	-0.14785600

Compound No. **5** [Neutral (Aq.)]

C	-3.03702900	1.15825000	0.10484600
C	-3.31332700	-0.22199300	-0.02423000
C	-2.29808800	-1.16493500	-0.11229300
C	-0.98061400	-0.69296800	-0.06159000
C	-0.67193900	0.69491600	0.05782800
C	-1.73245200	1.61615400	0.14462900
N	0.20373200	-1.38670400	-0.11123900
C	1.25604100	-0.47754900	-0.05633900
C	0.75433300	0.79978500	0.04843300
C	2.68266300	-0.76487600	0.03216700
N	3.55194800	0.18048800	-0.06997300
C	3.06285700	1.53284200	-0.39128200
C	1.71421800	1.93446900	0.23229500
C	3.13773200	-2.18027200	0.26962400
O	-4.64101500	-0.57077200	-0.05652400
H	-3.87129600	1.84654100	0.16897800
H	-2.51884200	-2.22234000	-0.21027800
H	-1.53405800	2.67788700	0.23843500
H	0.28849800	-2.38221700	-0.24393100
H	3.83879500	2.24535300	-0.10403500
H	2.97459600	1.58627200	-1.48642500
H	1.83234200	2.15815900	1.30211800
H	1.36188100	2.85526200	-0.24061400
H	4.22357800	-2.21253800	0.35177400
H	2.69632600	-2.58885900	1.18391400
H	2.83110700	-2.82963100	-0.55763500
H	-4.73094000	-1.52713000	-0.14615700

Compound No. **5** [Protonated (Gas)]

C	-3.04880000	1.16346200	0.09826600
C	-3.33559200	-0.22948600	-0.02032800
C	-2.32932600	-1.18521300	-0.10039100
C	-1.01370500	-0.72142500	-0.05603100
C	-0.69858600	0.67363200	0.04653800
C	-1.75752700	1.61337100	0.13262000
N	0.16155400	-1.42454400	-0.09172300
C	1.22231600	-0.51713400	-0.06589600
C	0.70160900	0.78281000	0.02744300
C	2.59176700	-0.79530100	0.04327500
N	3.43148500	0.23666300	-0.01168300
C	2.99877000	1.59625300	-0.38869300
C	1.63360200	1.93997400	0.21802300
C	3.13945100	-2.17575600	0.22528400
O	-4.64539300	-0.53890500	-0.04640700
H	-3.88863700	1.84383800	0.15639100
H	-2.56199600	-2.24095500	-0.18338500
H	-1.55011200	2.67312800	0.21861600
H	0.23373700	-2.41367500	-0.27066100
H	3.76708100	2.29523500	-0.06078300
H	2.94105000	1.63536500	-1.48283900
H	1.72505600	2.16006000	1.28930300
H	1.25820400	2.84751800	-0.25930300
H	4.20554400	-2.15944500	0.45430300
H	2.61981000	-2.69531300	1.03322700
H	3.00225900	-2.75586600	-0.69319600
H	-4.78681800	-1.48970900	-0.13048800
H	4.42281900	0.05510600	0.06324900

Compound No. **5** [Protonated (Aq.)]

C	-3.05164000	1.16561100	0.09830400
C	-3.33573100	-0.22329100	-0.01973800
C	-2.33143000	-1.17574000	-0.10197500
C	-1.01211700	-0.71013700	-0.05820000
C	-0.69798900	0.68235800	0.04757700
C	-1.75485400	1.61803800	0.13186400
N	0.15957700	-1.41233800	-0.10105400
C	1.21603500	-0.50762500	-0.06543500
C	0.71119600	0.78869000	0.03142300
C	2.59507100	-0.80099200	0.03904800
N	3.43192600	0.21693100	-0.02561200
C	3.01176500	1.58585000	-0.38419300
C	1.65021600	1.93944200	0.22142600
C	3.11891900	-2.18438000	0.23451500
O	-4.65671400	-0.54953900	-0.04557400
H	-3.88645900	1.85286100	0.15696400
H	-2.55859000	-2.23191100	-0.18892000
H	-1.54727900	2.67788900	0.21641400
H	0.23572200	-2.40735100	-0.24719100
H	3.78862200	2.26840900	-0.04594700
H	2.96115100	1.63343200	-1.47739100
H	1.74361300	2.16014700	1.29193200
H	1.28330500	2.84844300	-0.25860500
H	4.20271800	-2.19017900	0.34322500
H	2.66784500	-2.63873200	1.11959000
H	2.85294400	-2.80249800	-0.62806300
H	-4.77061900	-1.50470400	-0.13022100
H	4.42356800	0.03142200	0.05177600

Compound No. **6** [Neutral (Gas)]			
C	-3.49623700	0.82858300	0.07539400
C	-3.66901400	-0.56438100	-0.05907400
C	-2.58124400	-1.42280500	-0.13571800
C	-1.30463800	-0.85807200	-0.07167500
C	-1.10640300	0.54890900	0.05315400
C	-2.23076700	1.38994100	0.13114300
N	-0.06217300	-1.45556700	-0.10996600
C	0.91025400	-0.46656200	-0.04142700
C	0.30786400	0.76490200	0.05921200
C	2.35960300	-0.63597300	0.03321700
N	3.14650400	0.37179200	-0.07613400
C	2.55481200	1.68290100	-0.37776300
C	1.17527100	1.97183300	0.24487900
C	2.93835000	-2.00872300	0.26099300
H	-4.37079800	1.46639700	0.13314000
H	-2.72111100	-2.49355600	-0.23660600
H	-2.10837800	2.46291500	0.22986300
H	0.10773000	-2.43869600	-0.24030100
H	3.27515900	2.44647700	-0.07684300
H	2.47072000	1.74305400	-1.47277400
H	1.27545900	2.20175300	1.31520300
H	0.74797800	2.86206000	-0.22622800
H	4.02361500	-1.93745700	0.31815200
H	2.55767800	-2.45064300	1.18776500
H	2.67667200	-2.68613200	-0.56048800
H	-4.67214400	-0.97306000	-0.10219800
Compound No. **6** [Neutral (Aq.)]			
C	-3.49440100	0.82960800	0.07902800
C	-3.66555000	-0.56569900	-0.05862400
C	-2.57747000	-1.42420800	-0.13852900
C	-1.29952600	-0.85771700	-0.07408800
C	-1.10336200	0.55142000	0.05404700
C	-2.22904500	1.39327600	0.13481700
N	-0.06014100	-1.45456500	-0.11606300
C	0.91046100	-0.46688800	-0.04579300
C	0.31016200	0.76779800	0.05914900
C	2.35853000	-0.63952200	0.03454000
N	3.14634000	0.37246500	-0.07226800
C	2.55144100	1.68467200	-0.38368100
C	1.17743500	1.97442700	0.24574300
C	2.92577900	-2.01436800	0.26668300
H	-4.37011100	1.46595500	0.13874400
H	-2.71323600	-2.49468700	-0.24231500
H	-2.10716300	2.46624700	0.23487400
H	0.10695400	-2.44062100	-0.24116500
H	3.27053500	2.45302800	-0.09282700
H	2.45848800	1.73698700	-1.47820100
H	1.28250200	2.20217600	1.31589100
H	0.75038700	2.86602500	-0.22142900
H	4.01206900	-1.96213300	0.33062100
H	2.53240100	-2.45173000	1.18954300
H	2.65568900	-2.68848800	-0.55338000
H	-4.66864400	-0.97484800	-0.10178200

Compound No. **6** [Protonated (Gas)]

C	3.50165900	0.85100100	-0.08431800
C	3.68268300	-0.55067700	0.04406500
C	2.61644500	-1.42604000	0.12471500
C	1.33178800	-0.87162500	0.07122100
C	1.12467900	0.54061200	-0.04174100
C	2.24286100	1.40363200	-0.12815700
N	0.10621200	-1.48776800	0.10621800
C	-0.87874800	-0.50623900	0.06900800
C	-0.27050500	0.75114900	-0.02927500
C	-2.27067000	-0.68644700	-0.04336400
N	-3.03004500	0.39930400	0.01465700
C	-2.50176900	1.73109100	0.37962600
C	-1.11559100	1.97166800	-0.22690700
C	-2.90940900	-2.02420900	-0.23771100
H	4.37497800	1.48839200	-0.14458900
H	2.77194300	-2.49402400	0.21779400
H	2.10909100	2.47476900	-0.22061500
H	-0.03809400	-2.46947200	0.28365300
H	-3.22075500	2.47725600	0.04411500
H	-2.44574800	1.77255500	1.47343500
H	-1.18853200	2.19621300	-1.29876100
H	-0.67620100	2.85090000	0.24883300
H	-3.98261100	-1.94058100	-0.41118300
H	-2.45827900	-2.54520900	-1.08542300
H	-2.75874100	-2.64095900	0.65424000
H	4.69138000	-0.94519500	0.07759400
H	-4.03213400	0.29109800	-0.06819400

Compound No. **6** [Protonated (Aq.)]			
C	3.50647200	0.84905000	-0.08426700
C	3.68737500	-0.55137600	0.04387700
C	2.61667600	-1.42314400	0.12566300
C	1.33075800	-0.86663600	0.07297400
C	1.12571000	0.54427900	-0.04211700
C	2.24380300	1.40321000	-0.12752400
N	0.10702600	-1.47931900	0.11318400
C	-0.87254600	-0.49883500	0.06681400
C	-0.27635200	0.75512000	-0.03345800
C	-2.27556900	-0.68864300	-0.04208200
N	-3.02907400	0.38695900	0.02575100
C	-2.50928100	1.72524000	0.37684000
C	-1.12546600	1.97259200	-0.22925500
C	-2.89940400	-2.02780300	-0.24396100
H	4.37913200	1.48821500	-0.14542900
H	2.76372200	-2.49206700	0.21974100
H	2.10941400	2.47444100	-0.21988500
H	-0.04390700	-2.46535000	0.26376600
H	-3.23532700	2.45887400	0.03272500
H	-2.45870600	1.77348600	1.46961500
H	-1.20141200	2.19785800	-1.30012600
H	-0.69126600	2.85223000	0.24923600
H	-3.96963200	-1.94804600	-0.43009200
H	-2.42655000	-2.54453300	-1.08149300
H	-2.74739800	-2.63754700	0.65188300
H	4.69520300	-0.94885300	0.07684300
H	-4.03198800	0.27948600	-0.06034900
Compound No. **7** [Neutral (Gas)]			
C	3.76776100	0.43640800	0.05568700
C	3.59988300	-0.93601800	-0.13101500
C	2.32183100	-1.47913600	-0.19894700
C	1.18892900	-0.66411600	-0.07569200
C	1.36120800	0.72041000	0.11904800
C	2.64599400	1.25529700	0.17728000
N	-0.08160600	-1.26190100	-0.17521400
C	-1.07985900	-0.44988300	-0.22201800
N	-1.04249100	0.91373400	-0.16978900
C	0.14458600	1.60321500	0.31569500
C	-2.52661000	-0.90450000	-0.28718100
C	-3.29316700	0.38813300	-0.58176000
C	-2.37312100	1.50621900	-0.05781800
H	4.76154200	0.86626300	0.10434800
H	4.46590900	-1.58129100	-0.22811100
H	2.16972800	-2.54119000	-0.35240100
H	2.77328500	2.32432500	0.32265200
H	0.26096900	2.54947300	-0.22454100
H	0.01606500	1.85862600	1.38267900
H	-3.43441300	0.49368500	-1.65980000
H	-4.27131200	0.38375900	-0.10147700
H	-2.43766600	2.42396100	-0.64917200
H	-2.59156200	1.75912300	0.98888600
H	-2.66594000	-1.67366900	-1.05210700
O	-2.92919800	-1.40092800	0.99368500
H	-2.28894700	-2.07464200	1.25491800

Compound No. **7** [Neutral (Aq.)]			
C	3.78301500	0.42647000	0.04326400
C	3.61348200	-0.94708300	-0.14027900
C	2.33318700	-1.48939900	-0.19607600
C	1.19806900	-0.67505200	-0.06348000
C	1.37409900	0.71063600	0.12911400
C	2.66063000	1.24510600	0.17368800
N	-0.07228500	-1.27645800	-0.15044700
C	-1.07808200	-0.46153000	-0.17178500
N	-1.03453200	0.89176500	-0.10327300
C	0.16284100	1.59951900	0.32974300
C	-2.53004300	-0.89749800	-0.27681100
C	-3.25439800	0.40406500	-0.65813700
C	-2.35738600	1.51595600	-0.09062800
H	4.77693200	0.85685000	0.08361900
H	4.47782200	-1.59368600	-0.24378700
H	2.18738600	-2.55345700	-0.34646800
H	2.78753800	2.31402900	0.31680900
H	0.26055800	2.52661500	-0.24422500
H	0.05993700	1.88915000	1.38801600
H	-3.30427800	0.48454500	-1.74670500
H	-4.27139000	0.43664100	-0.26635900
H	-2.35768100	2.41951100	-0.70439700
H	-2.63598600	1.79798400	0.93237400
H	-2.65212700	-1.68822600	-1.02045500
O	-2.90063200	-1.38469600	1.02300900
H	-3.80842700	-1.70667600	0.97187900
Compound No. **7** [Protonated (Gas)]			
C	3.79165800	0.41800100	0.13358200
C	3.62143400	-0.95309900	-0.04887500
C	2.34247300	-1.48344900	-0.18072800
C	1.24301400	-0.63075100	-0.12513800
C	1.39286500	0.74590500	0.05766900
C	2.68172400	1.25888400	0.18586400
N	-0.07167200	-1.15736400	-0.26342200
C	-1.13694900	-0.37789800	-0.22088400
N	-1.06269300	0.92360400	-0.06588700
C	0.19067900	1.66828400	0.13017300
C	-2.57230400	-0.86034200	-0.28213400
C	-3.35610900	0.46561400	-0.41911100
C	-2.38741200	1.56846900	0.04252800
H	4.78636100	0.83366200	0.23532400
H	4.48095700	-1.61039400	-0.08973500
H	2.20059900	-2.54905900	-0.32520300
H	2.82120100	2.32502500	0.32932500
H	0.24703300	2.44889200	-0.63574200
H	0.13845000	2.16754000	1.10444000
H	-3.63381100	0.62084200	-1.46314600
H	-4.26889200	0.44194200	0.17516300
H	-2.41460200	2.45563000	-0.59218900
H	-2.54528200	1.87743900	1.07933800
H	-2.72204700	-1.51284800	-1.14910200
O	-2.77562800	-1.57029900	0.92854700
H	-3.60597000	-2.05922600	0.88699100
H	-0.20485000	-2.15844300	-0.31634900

Compound No. **7** [Protonated (Aq.)]			
C	3.79416100	0.42038200	0.12795000
C	3.62502100	-0.95093500	-0.06020600
C	2.34608100	-1.48445000	-0.18592100
C	1.24219900	-0.63600600	-0.11960500
C	1.39237900	0.74109200	0.06710200
C	2.68094100	1.25715800	0.18950200
N	-0.06673200	-1.16465500	-0.24739400
C	-1.13352600	-0.38306800	-0.20522800
N	-1.05983600	0.91501000	-0.04628900
C	0.19034300	1.66179300	0.14166800
C	-2.56919200	-0.85984700	-0.27664200
C	-3.33844900	0.46484200	-0.46327000
C	-2.38365400	1.56603600	0.02468300
H	4.78836100	0.83890800	0.22581500
H	4.48540000	-1.60697400	-0.11021200
H	2.20300200	-2.54848900	-0.33479000
H	2.81352200	2.32361700	0.33492900
H	0.24376500	2.43718300	-0.62814600
H	0.14311700	2.16483000	1.11275200
H	-3.56168100	0.60590700	-1.52165600
H	-4.27685400	0.44986700	0.08872400
H	-2.38586000	2.44908000	-0.61457600
H	-2.56725900	1.87884800	1.05532500
H	-2.70802400	-1.54053400	-1.11944000
O	-2.82227700	-1.52897400	0.95539300
H	-3.59642000	-2.09343200	0.84442600
H	-0.19470200	-2.16149400	-0.36072000

Compound No. **8** [Neutral (Gas)]			
C	3.74382800	0.23863700	0.09976000
C	3.57136000	-1.14690000	-0.05683300
C	2.30494200	-1.69245100	-0.16654300
C	1.16876100	-0.86336700	-0.12126700
C	1.34792100	0.53242700	0.03630700
C	2.63914600	1.07044100	0.14531100
N	-0.08713700	-1.44895400	-0.23445400
C	-1.09001500	-0.64674200	-0.18599900
N	-1.02316800	0.72164800	-0.05517200
C	0.17795200	1.42121300	0.08212000
C	-2.55119500	-1.05139200	-0.24286000
C	-3.24634400	0.26441100	-0.64301200
C	-2.34459100	1.36656800	-0.05982700
H	4.74135200	0.65360800	0.18509000
H	4.43975500	-1.79555400	-0.09072000
H	2.15443300	-2.75862900	-0.28505800
H	2.74096900	2.14230700	0.26488200
H	-3.27401500	0.34539900	-1.73366700
H	-4.26872300	0.31388700	-0.26758700
H	-2.30627300	2.27565600	-0.65860300
H	-2.62200800	1.64010600	0.96102900
H	-2.69456400	-1.85667700	-0.96678400
O	-2.91531900	-1.48231500	1.07204700
H	-3.69207900	-2.04640600	1.00938400
O	0.19315500	2.63486200	0.21376300

Compound No. **8** [Neutral (Aq.)]			
C	3.74566500	0.23976400	0.09842200
C	3.57304300	-1.14707200	-0.05459800
C	2.30599700	-1.69189800	-0.16365100
C	1.16936100	-0.86183000	-0.12189800
C	1.34738100	0.53477700	0.03277800
C	2.64089500	1.07148200	0.14156700
N	-0.08763700	-1.44381500	-0.23411000
C	-1.09392800	-0.64012900	-0.19548900
N	-1.02270200	0.72649100	-0.06409900
C	0.17515000	1.41506400	0.07906500
C	-2.55284800	-1.04926900	-0.25096600
C	-3.26208100	0.26709800	-0.61190700
C	-2.34805500	1.37382700	-0.06096100
H	4.74291700	0.65477600	0.18242400
H	4.44091200	-1.79579300	-0.08695800
H	2.16174000	-2.75932600	-0.28082300
H	2.75222600	2.14238500	0.25864400
H	-3.33231300	0.35036300	-1.69904500
H	-4.27003900	0.31258100	-0.20027600
H	-2.32128900	2.26690600	-0.68269500
H	-2.60358000	1.66803300	0.95925400
H	-2.70791000	-1.83419500	-0.99395300
O	-2.88408100	-1.53157100	1.05806900
H	-3.78171400	-1.88366100	1.02945400
O	0.19507400	2.63511000	0.22283700
Compound No. **8** [Protonated (Gas)]			
C	3.76420500	0.23988100	0.10955500
C	3.58551400	-1.14299600	-0.02841900
C	2.31414100	-1.68763800	-0.14597400
C	1.21287600	-0.83247400	-0.12346700
C	1.37398000	0.55908500	0.01511300
C	2.66714800	1.08599700	0.13270200
N	-0.09409800	-1.34217000	-0.24338800
C	-1.15304400	-0.55657000	-0.22278600
N	-1.04355600	0.75619000	-0.09128800
C	0.20988500	1.45443300	0.05522000
C	-2.59940000	-1.00543300	-0.27204900
C	-3.34550100	0.34018100	-0.44230900
C	-2.35971500	1.43888500	-0.00483300
H	4.76333400	0.64735600	0.19944300
H	4.44711400	-1.79934700	-0.04388300
H	2.17861000	-2.75816400	-0.25199700
H	2.78113000	2.15735300	0.24143200
H	-3.61775200	0.47401400	-1.49090800
H	-4.26093000	0.35239100	0.14845700
H	-2.35144500	2.31148700	-0.65524500
H	-2.50239500	1.77560300	1.02288300
H	-2.76700300	-1.67394400	-1.12398300
O	-2.80979300	-1.68226800	0.95530000
H	-3.66605500	-2.12682600	0.94422200
O	0.20660500	2.64808100	0.19550900
H	-0.23605600	-2.34466600	-0.29067600

Compound No. **8** [Protonated (Aq.)]

C	3.76204900	0.24672800	0.10084000
C	3.58739400	-1.13723700	-0.04527600
C	2.31926700	-1.68653500	-0.15483000
C	1.21250600	-0.83613300	-0.11626400
C	1.36927400	0.55506600	0.02832300
C	2.66216900	1.08651700	0.13764000
N	-0.08618900	-1.35021200	-0.22458200
C	-1.14417100	-0.57002800	-0.20389700
N	-1.03653800	0.74900400	-0.07587800
C	0.19942000	1.43854200	0.06985400
C	-2.59197300	-1.01113300	-0.25881300
C	-3.31747600	0.32649900	-0.51722300
C	-2.35840800	1.42696100	-0.03709500
H	4.76001900	0.65797300	0.18429600
H	4.45155600	-1.78933700	-0.07375500
H	2.18304400	-2.75486000	-0.26884700
H	2.77657800	2.15687900	0.24915700
H	-3.50249900	0.43336100	-1.58698500
H	-4.27278900	0.35355800	0.00382100
H	-2.32854000	2.29640800	-0.68950100
H	-2.54444400	1.75620500	0.98554800
H	-2.74569900	-1.72937500	-1.06692600
O	-2.86284000	-1.60246400	1.00610000
H	-3.63440900	-2.17511400	0.91960800
O	0.20479700	2.64243500	0.21119500
H	-0.21968300	-2.35249200	-0.31110800
